# The Machado–Joseph disease deubiquitylase ataxin‐3 interacts with LC3C/GABARAP and promotes autophagy

**DOI:** 10.1111/acel.13051

**Published:** 2019-10-17

**Authors:** Laura K. Herzog, Éva Kevei, Ricardo Marchante, Claudia Böttcher, Christian Bindesbøll, Alf Håkon Lystad, Annika Pfeiffer, Maria E. Gierisch, Florian A. Salomons, Anne Simonsen, Thorsten Hoppe, Nico P. Dantuma

**Affiliations:** ^1^ Department of Cell and Molecular Biology Karolinska Institutet Stockholm Sweden; ^2^ Institute for Genetics and CECAD Research Center University of Cologne Cologne Germany; ^3^ Department of Molecular Medicine Faculty of Medicine Institute of Basic Medical Sciences and Centre for Cancer Cell Reprogramming Institute of Clinical Medicine University of Oslo Oslo Norway; ^4^ Present address: School of Biological Sciences University of Reading Reading UK

**Keywords:** ataxin‐3, *atx‐3*, autophagy, *Caenorhabditis elegans*, DUB, ubiquitin

## Abstract

The pathology of spinocerebellar ataxia type 3, also known as Machado‐Joseph disease, is triggered by aggregation of toxic ataxin‐3 (ATXN3) variants containing expanded polyglutamine repeats. The physiological role of this deubiquitylase, however, remains largely unclear. Our recent work showed that ATX‐3, the nematode orthologue of ATXN3, together with the ubiquitin‐directed segregase CDC‐48, regulates longevity in *Caenorhabditis elegans*. Here, we demonstrate that the long‐lived *cdc‐48.1; atx‐3* double mutant displays reduced viability under prolonged starvation conditions that can be attributed to the loss of catalytically active ATX‐3. Reducing the levels of the autophagy protein BEC‐1 sensitized worms to the effect of ATX‐3 deficiency, suggesting a role of ATX‐3 in autophagy. In support of this conclusion, the depletion of ATXN3 in human cells caused a reduction in autophagosomal degradation of proteins. Surprisingly, reduced degradation in ATXN3‐depleted cells coincided with an increase in the number of autophagosomes while levels of lipidated LC3 remained unaffected. We identified two conserved LIR domains in the catalytic Josephin domain of ATXN3 that directly interacted with the autophagy adaptors LC3C and GABARAP in vitro. While ATXN3 localized to early autophagosomes, it was not subject to lysosomal degradation, suggesting a transient regulatory interaction early in the autophagic pathway. We propose that the deubiquitylase ATX‐3/ATXN3 stimulates autophagic degradation by preventing superfluous initiation of autophagosomes, thereby promoting an efficient autophagic flux important to survive starvation.

## Graphical abstract

The deubiquitylase ataxin‐3 has been linked to longevity in *Caenorhabditis elegans*, a phenotype that typically coincides with enhanced autophagy. The longevity phenotype comes at the expense of an increased sensitivity to starvation, indicative for a defect in autophagy. This study reveals a novel, stimulatory role of ataxin‐3 in autophagy by preventing superfluous induction of autophagosomes.

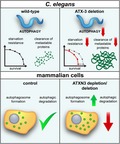

## 1 INTRODUCTION

Ataxin‐3 (ATXN3), which is the founding member of the family of Josephin domain‐containing deubiquitylases (DUBs), plays a critical role in the maintenance of protein homeostasis and stress resistance (Burnett & Pittman, 2005; Durcan et al., 2011; Scaglione et al., 2011; Wang, Li, & Ye, 2006; Zhong & Pittman, 2006). Abnormal expansion of a stretch of glutamine residues in the C‐terminus of ATXN3 is causative for spinocerebellar ataxia type 3 (SCA3), also known as Machado–Joseph disease, the most common hereditary type of ataxia (Kawaguchi et al., 1994). While mutant ATXN3 causes neuronal demise through a predominant, toxic gain‐of‐function mechanism typically associated with polyglutamine (polyQ) disorders, wild‐type ATXN3 has neuroprotective properties and can reduce the toxicity caused by polyglutamine proteins in cellular and *Drosophila* models (Tsou et al., 2015; Warrick et al., 2005). Hence, a loss of its protective function may contribute to the disease, consistent with a general role of ATXN3 in maintaining protein homeostasis.

Interestingly, simultaneous inactivation of ATX‐3, and the ubiquitin‐selective segregase CDC‐48.1 in the nematode *Caenorhabditis elegans* (Olszewski, Williams, Dong, & Martin, 2019), results in extended lifespan (Kuhlbrodt et al., 2011). This synergistic effect on the longevity of *cdc‐48.1; atx‐3* double mutants depends on the insulin/insulin‐like growth factor‐1 signaling (IIS) pathway (Kuhlbrodt et al., 2011). Aging is typically accompanied by a gradual decline in the functionality of autophagy, and conversely, genetic or dietary stimulation of autophagy promotes longevity in various model organisms (Leidal, Levine, & Debnath, 2018). Hence, there appears to be a positive correlation between lifespan and the functionality of autophagy, which is also triggered by IIS (Melendez et al., 2003).

Another emerging theme is the involvement of proteins linked to age‐related neurodegenerative disorders in the autophagic degradation of aberrant proteins and organelles (Menzies, Fleming, & Rubinsztein, 2015). Dysfunction of neurodegeneration‐associated proteins involved in autophagy may lead to impaired autophagy, which has been proposed to contribute to the pathophysiology in various neurodegenerative disorders (Ashkenazi et al., 2017; Ju et al., 2009; Martinez‐Vicente et al., 2010; Rui et al., 2015; Tresse et al., 2010). Thus, ATX‐3/ATXN3 is linked to both longevity and neurodegenerative disorders, which are associated with increased and impaired autophagy, respectively.

Autophagy can be triggered by a variety of stressors, such as the presence of cytotoxic protein aggregates, to ensure cell survival, while also regulating nutrient recycling during periods of nutrient deprivation. The initiation, formation, and elongation of the autophagosomal membrane is regulated by phosphatidylethanolamine (PE) conjugation and autophagosomal anchoring of six highly related ubiquitin‐like modifiers belonging to the Atg8 family: LC3A, LC3B, LC3C, GABARAP, GABARAPL1, and GABARAPL2. LC3s and GABARAPs play critical roles in facilitating the recruitment of cargo receptors and other autophagy proteins to autophagosomes by directly interacting with these proteins through conserved LC3‐interacting regions (LIRs) (Johansen & Lamark, 2011). After fusion with lysosomes, the fraction of LC3/GABARAP localized on the luminal side of autophagic vesicles is degraded in the lysosomes together with cargo receptors and substrates, while LC3/GABARAP present on the cytosolic face is released by Atg4 protease (Abreu et al., 2017).

Despite the well‐established involvement of ubiquitin ligases in autophagy, the role of DUBs in this process has only recently gained interest (Clague & Urbe, 2017). Beclin‐1 has been shown to be a prominent substrate of various DUBs, including USP10, USP13 (Liu et al., 2011), USP9X (Elgendy et al., 2014), and ATXN3 (Ashkenazi et al., 2017). As a core component of the class III PI3‐kinase/Vps34 complex, beclin‐1 supports the induction of autophagy by stimulation of Vps34‐dependent generation of phosphatidylinositol‐3‐phosphate. Stabilization of beclin‐1 by ATXN3 has been proposed to promote lipidation of LC3 and autophagy (Ashkenazi et al., 2017).

Here, we show that the long‐lived *cdc‐48.1; atx‐3* double mutant displays increased sensitivity to nutrient starvation and impaired ability to clear aggregation‐prone proteins, two phenotypes that are indicative for defective autophagy. In agreement with a new physiological role of ATXN3 in autophagy, we show that ATXN3 is an LC3C/GABARAP‐interacting DUB that regulates the formation of autophagosomes independent from its stabilizing effect on beclin‐1 (Ashkenazi et al., 2017). Our study implicates a conserved regulatory role for ATX‐3/ATXN3 in autophagy and shows that increased lifespan can come at the expense of a reduced ability to maintain organismal protein homeostasis under stress conditions, such as starvation.

## 2 RESULTS

### 2.1 Autophagy is impaired in ATX‐3‐deficient worms

**Figure 1:**
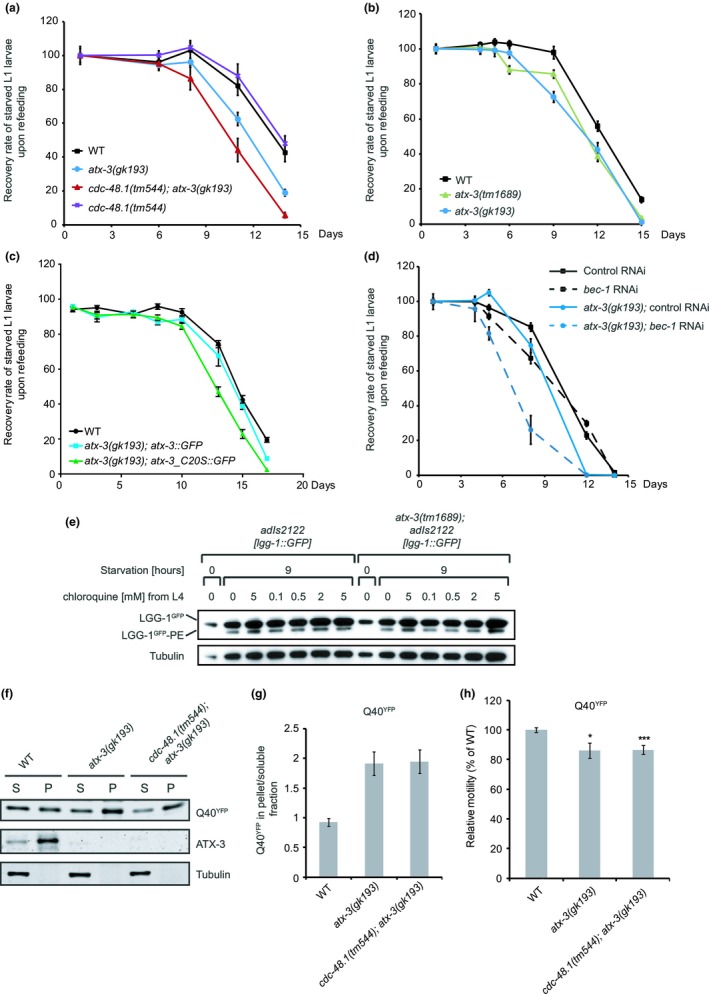
Autophagy is impaired in *atx‐3* deletion worms. (a) Survival rate upon refeeding of starved L1 larvae of the long‐lived strain *cdc‐48.1(tm544); atx‐3(gk193)* or the single‐deletion strains *atx‐3(gk193)* and *cdc‐48.1(tm544)* compared with wild‐type worms. (b) Starved L1 larvae of the *atx‐3* deletion strains *atx‐3(tm1689)* and *atx‐3(gk193)* show decreased survival rate upon refeeding compared with wild‐type worms. (c) The decreased survival of the *atx‐3(gk193)* mutant strain can be rescued by introduction of GFP‐tagged wild‐type ATX‐3 protein but not the catalytic inactive mutant C20S. (d) Reduction in *bec‐1* levels by RNAi further decreases the survival of *atx‐3(gk193)* mutant worms upon starvation. (e) Western blot analysis of autophagy protein LGG‐1 in total worm lysates of day 1 adult well‐fed worms or worms starved for 9 hr. Worms were treated with the lysosomal inhibitor chloroquine at the indicated concentration from L4 stage (performed in duplicates for treatment with 5 mM chloroquine). Immunoblotting was performed using anti‐GFP and anti‐tubulin antibodies. (f) Total worm lysates of mutant and wild‐type worms expressing Q40‐YFP aggregation‐prone protein in body‐wall muscle cells have been subjected to Western blot analysis. The Western blot shows the amounts of Q40::YFP and ATX‐3 protein in the PBS‐soluble (S) and PBS‐insoluble (pellet, P; only dissolved in SDS‐containing buffer) fraction of total worm lysates in WT, *atx‐3(gk193)*, and *cdc‐48.1*(*tm1544*); *atx‐3*(*gk193*) mutants. Western blot analysis was done using anti‐GFP and anti‐ATX‐3 antibodies. (g) Quantification of relative band intensities of Q40‐YFP in soluble and pellet fraction was done. Data are presented as mean value and *SD* derived from two independent experiments. (h) Relative motility of indicated strains expressing aggregation‐prone Q40‐YFP protein in body‐wall muscle cells (Student's *t* test, unpaired, two‐tailed, *n* ≥ 20, *p ≤ .05, ***p ≤ .001)

Given the correlation between lifespan and autophagy, we explored the functional status of autophagy in long‐lived *cdc‐48.1; atx‐3* double mutant worms. To this end, we examined the survival of starved L1 larvae, a metabolic challenge that strongly depends on functional autophagy (Kovacs & Zhang, 2010). When worms hatch in the absence of food, larvae arrest at the L1 diapause and can survive for several weeks, from which they recover and resume growth upon refeeding. We found that the *cdc‐48.1; atx‐3* deletion mutant had a significantly decreased recovery rate and reduced viability in comparison with wild‐type worms, indicative of a defect in the autophagy–lysosome pathway (Figure 1a, Table S1). ATXN3 and p97 (also known as valosin‐containing protein (VCP)), which are the human orthologues of ATX‐3 and CDC‐48, respectively, have both been implicated in autophagy (Ashkenazi et al., 2017; Ju et al., 2009; Tresse et al., 2010). To clarify to what extent the increased sensitivity could be attributed to ATX‐3 or CDC‐48, we next evaluated the survival of *atx‐3* or *cdc‐48.1* single‐deletion strains. While the deletion of CDC‐48 did not increase the sensitivity of larvae to starvation (Figure 1a, Table S1), the two independent *atx‐3* deletion alleles *tm1689* and *gk193* phenocopied the starvation sensitivity of the *cdc‐48.1; atx‐3* double mutant (Figure 1b, Table S2). Importantly, the decreased survival of ATX‐3‐deficient worms upon starvation could be rescued by reintroduction of GFP‐tagged wild‐type ATX‐3 but not by the catalytically inactive ATX‐3^C20S^ mutant (Figure 1c, Table S3).

While autophagy promotes the survival of L1 larvae during starvation, its excessive activation or misregulation is not beneficial but rather increases the mortality of starving worms (Kang, You, & Avery, 2007). To analyze whether reduced or increased autophagy is accountable for the decreased survival of *atx‐3* deletion worms, we curtailed autophagy through silencing of *bec‐1*, the worm orthologue of beclin‐1. We found that the partial depletion of *bec‐1* did not rescue but further reduced the survival of the mutant worms, implying that the deletion of *atx‐3* results in a reduced activity of autophagy (Figure 1d, Table S4). Moreover, it suggests that it reduces autophagy activity independent of the BEC‐1 complex, which was further supported by the observation that the levels of lipidated GFP‐LGG‐1, the nematode orthologue of LC3/GABARAP, remained unaffected upon deletion of ATX‐3 (Figure 1e). Similarly, we observed that the partial depletion of *unc‐51*, the nematode orthologue of ULK1, which is the upstream Ser/Thr protein kinase involved in the activation of autophagy in *C. elegans*, further aggravated the mortality of *atx‐3* mutant worms (Figure S1A, Table S5).

Another process that strongly depends on functional autophagy and that is of direct relevance for polyQ diseases is the clearance of aggregation‐prone proteins (Ravikumar et al., 2004). Both wild‐type and ATX‐3‐deficient worms developed visible inclusions upon muscle‐specific expression of an aggregation‐prone polyQ repeat (Morley, Brignull, Weyers, & Morimoto, 2002) (Figure S1B). Biochemical analysis showed an increase in SDS‐insoluble polyQ‐YFP aggregates in the *atx‐3*‐deficient strain that was comparable to the *cdc‐48.1; atx‐3* double mutant, consistent with reduced clearance of protein aggregates (Figure 1f,g). PolyQ‐based aggregation in the muscle triggered reduced motility of *atx‐3* and *cdc‐48.1; atx‐3* mutant worms compared with wild‐type worms (Figure 1h). Combined, these data suggest a stimulatory role of ATX‐3 in autophagy in a multicellular organism relevant for the cellular stress response to starvation and protein aggregation.

### 2.2 ATXN3 depletion impairs autophagic degradation of long‐lived proteins and increases the number of autophagosomes

**Figure 2:**
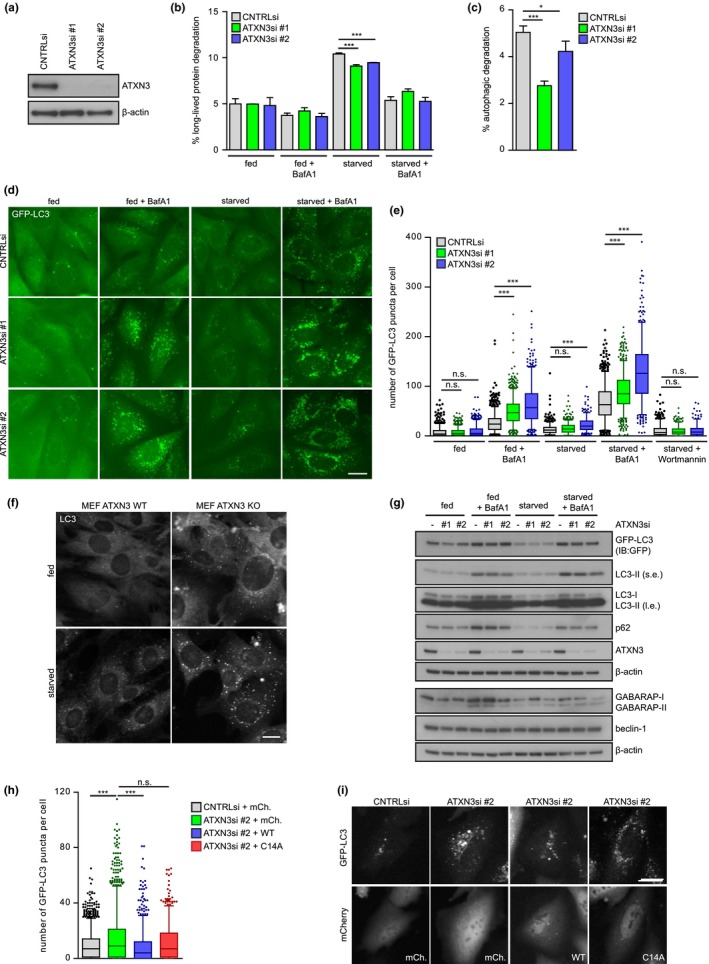
ATXN3 depletion increases the number of autophagosomes and impairs degradation of long‐lived proteins. (a) Knockdown efficiency of two different siRNAs targeting ATXN3 (ATXN3si #1, ATXN3si #2) in HOS GFP‐LC3 cells. (b) Long‐lived protein degradation assay in U2OS cells depleted of ATXN3 and treated as indicated. Percentage of long‐lived protein degradation was quantified. Data are presented as mean ± *SD* of three independent experiments, each performed in technical duplicates. *p ≤ .05, **p ≤ .01, ***p ≤ .001 (one‐way ANOVA). (c) Percentage of long‐lived protein degradation by autophagy was quantified as the bafilomycin A1‐sensitive fraction of degradation during starvation in (b); *p ≤ .05, **p ≤ .01, ***p ≤ .001 (one‐way ANOVA). (d) Representative micrographs of HOS GFP‐LC3 cells depleted of ATXN3 using two different siRNAs (ATXN3si #1, ATXN3si #2). Cells were treated as indicated and imaged live using an automated widefield microscope. Scale bar: 20 μm. (e) Quantification of GFP‐LC3 puncta per cell upon ATXN3 depletion in HOS GFP‐LC3 cells. Data are presented as box plot with median and 5–95 percentile of at least two independent experiments, *n* ≥ 360, *p ≤ .05, **p ≤ .01, ***p ≤ .001 (Kruskal–Wallis test). (f) Representative micrographs of endogenous LC3B in wild‐type or ATXN3 knockout (KO) mouse embryonic fibroblasts (MEFs) treated as indicated for 4 hr. Scale bar: 20 μm (g) Western blot analysis of autophagy proteins in HOS GFP‐LC3 upon siRNA‐mediated depletion of ATXN3. Cells were treated as indicated for 4 hr and analyzed by immunoblotting using the indicated antibodies (s.e.: short exposure; l.e.: long exposure). (h) Quantification of GFP‐LC3 puncta per cell upon ATXN3 depletion using ATXN3si #2 and transient overexpression of mCherry‐C1 (mCh.), mCherry‐ATXN3 WT (WT), or mCherry‐ATXN3 C14A (C14A) in HOS GFP‐LC3 cells. Cells were starved for 4 hr in the presence of 100 nM bafilomycin A1. Data are presented as box plot with median and 5–95 percentile of four independent experiments, *n* ≥ 700, *p ≤ .05, **p ≤ .01, ***p ≤ .001 (Kruskal–Wallis test). (i) Representative micrographs of HOS GFP‐LC3 cells depleted of ATXN3 using ATXN3si #2 and transfected with mCherry‐C1 (mCh.), mCherry‐ATXN3 WT (WT), or mCherry‐ATXN3 C14A (C14A). Cells were imaged live using an automated widefield microscope. Intensities in micrographs showing mCherry signal of WT and C14A samples were rescaled. Scale bar: 20 μm

To explore whether human ATXN3 is involved in autophagy, we analyzed the autophagic flux directly by determining the fraction of long‐lived intracellular proteins that were either degraded by basal or starvation‐induced autophagy in control or ATXN3‐depleted cells. We used two different ATXN3‐specific siRNAs that both efficiently depleted ATXN3 (Figure 2a). Proteins were metabolically labeled, followed by a 16‐hr chase to allow degradation of short‐lived proteins. Degradation of long‐lived proteins during a 4‐hr starvation period was monitored by quantification of free radiolabeled amino acids (Klionsky et al., 2016). This analysis revealed that the depletion of ATXN3 with two independent siRNAs caused a significant reduction in degradation of long‐lived proteins in starved cells (Figure 2b), which corresponded to an estimated 20%–50% reduction in autophagic flux based on the difference in degradation of long‐lived proteins in control and ATXN3‐depleted cells during starvation compared with starvation in the presence of bafilomycin A1 (Figure 2c).

GFP‐LC3 shows diffuse fluorescence under normal conditions but localizes to autophagosomes when autophagy is induced by nutrient starvation, which can be quantified by fluorescence microscopy analysis of the number of fluorescent puncta. Surprisingly, the depletion of ATXN3 with either siRNA resulted in a significant increase in GFP‐LC3 puncta when lysosomal degradation was blocked by bafilomycin A1 treatment under basal and nutrient‐starved conditions (Figure 2d,e). This increase suggests that more autophagic structures were formed in ATXN3‐depleted cells during the treatment. To validate this finding, we stained similarly treated wild‐type and ATXN3 knockout murine embryonic fibroblasts (Schmitt et al., 2007; Weishaupl et al., 2019) for endogenous LC3, which confirmed elevated levels of both basal and starvation‐induced LC3 puncta in ATXN3 knockout cells (Figure 2f, Figure S2A,B). No increase in LC3 foci was observed when ATXN3‐depleted cells were starved in the presence of the phosphatidylinositol‐3‐kinase (PI3K) inhibitor wortmannin, indicating that ATXN3 acts in the canonical autophagy–lysosome pathway downstream of PI3K (Figure 2e, Figure S2C). The increase in GFP‐LC3 puncta in ATXN3‐depleted cells was not accompanied by a detectable increase in the levels of lipidated GFP‐LC3 (Figure 2g) or endogenous lipidated LC3/GABARAP (Figure 2g, Figure S2D,E). This observation suggests that the accumulation of GFP‐LC3 puncta in ATXN3‐depleted cells does not reflect a change in the levels of autophagosome‐associated LC3.

Despite its ability to increase the number of autophagosomes, the depletion of ATXN3 did not increase the autophagic flux as measured using double‐tagged mRFP‐GFP‐LC3 (Figure S3A). We, however, consistently detected increased mRFP and GFP intensities in ATXN3‐depleted samples during starvation, both in the presence or in the absence of bafilomycin A1 treatment compared with controls, confirming the increase in autophagosomes detected by microscopy (Figure S3B‐E). Notably, the levels of beclin‐1 were not altered in ATXN3‐depleted GFP‐LC3 HOS cells (Figure 2g), HeLa cells (Figure S2D), or U2OS cells (Figure S2E), excluding that effects on autophagy were caused by enhanced proteasomal degradation of beclin‐1, which has recently been identified as an ATXN3 substrate (Ashkenazi et al., 2017).

The increase in GFP‐LC3 puncta upon ATXN3 depletion was rescued by transient overexpression of wild‐type but not catalytically inactive mCherry‐ATXN3, confirming that the observed effect is specific for ATXN3 (Figure 2h,i, Figure S4). Furthermore, in line with our observations in *C. elegans*, these data suggest that ATXN3 requires its DUB activity to support autophagy.

### 2.3 ATXN3 acts upstream of p97/VCP

**Figure 3:**
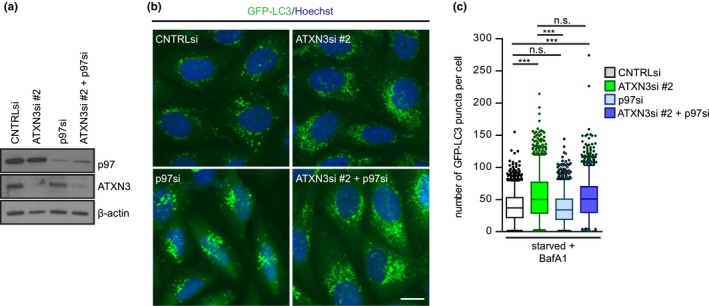
ATXN3 acts upstream of p97/VCP in the autophagy pathway. (a) Knockdown efficiencies of siRNA oligos targeting ATXN3 and/or p97/VCP after 48 hr. (b) Representative micrographs of HOS GFP‐LC3 cells depleted of ATXN3, p97/VCP, or both using the indicated siRNAs. Cells were starved in the presence of 100 nM bafilomycin A1 for 4 hr. Scale bar: 20 μm. (c) Quantification of GFP‐LC3 puncta in (b). Data are shown as box plot with median and 5–95 percentile of two independent experiments, *n* > 1,200, *p ≤ .05, **p ≤ .01, ***p ≤ .001 (Kruskal–Wallis test)

The ATXN3 interactor p97/VCP, the human orthologue of CDC‐48, has been linked to the maturation of autophagosomes by a yet‐to‐be determined mechanism (Ju et al., 2009; Tresse et al., 2010). In order to assess whether the effect of ATXN3 is epistatic with p97/VCP depletion, we either depleted ATXN3, p97/VCP, or both simultaneously, and analyzed the accumulation of LC3 puncta. Despite the fact that both p97/VCP and ATXN3 depletion had a striking effect on the localization of GFP‐LC3 foci, the distribution pattern was rather different with a more perinuclear localization of LC3 in p97/VCP‐depleted cells (Figure 3a,b). The number of LC3 puncta was not affected upon p97/VCP depletion, in contrast to the significantly increased number of GFP‐LC3 puncta in ATXN3‐depleted cells (Figure 3b,c). In cells that had been co‐depleted of p97/VCP and ATXN3 (Figure 3a‐c), the number of GFP‐LC3 puncta as well as their distribution pattern was similar to what was observed when ATXN3 alone was depleted, suggesting that ATXN3 acts upstream of p97/VCP. These observations are in line with a stimulatory role of ATXN3 in autophagy through regulation of an event that precedes the maturation step stimulated by p97/VCP.

### 2.4 ATXN3 interacts directly with GABARAP and LC3C

**Figure 4:**
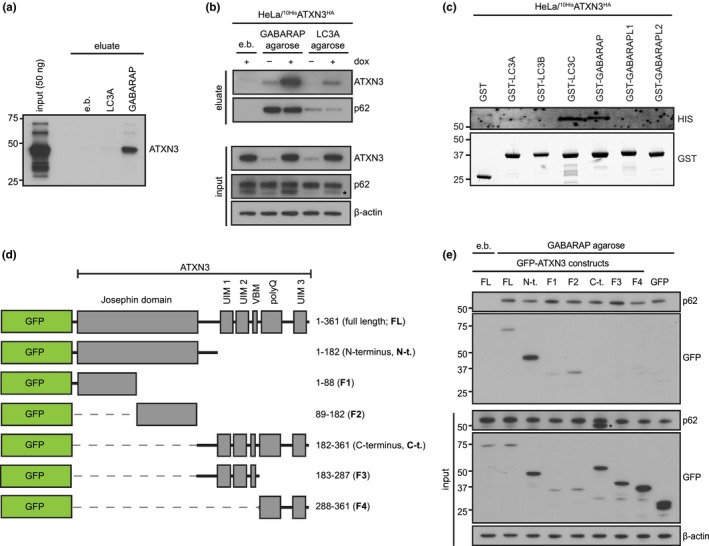
ATXN3 directly interacts with GABARAP and LC3C. (a) Purified, recombinant ATXN3 was incubated with blocked agarose beads (empty beads, e.b.) or recombinant GABARAP/LC3A immobilized on agarose beads. Binding was analyzed by immunoblotting. (b) Cell lysates from HeLa cells expressing doxycycline (dox)‐inducible ^10xHis^ATXN3^HA^ were incubated with blocked agarose beads (e.b.) or recombinant GABARAP/LC3A immobilized on agarose beads. Binding of endogenous ATXN3 and ^10xHis^ATXN3^HA^ was analyzed by immunoblotting with the indicated antibodies; * indicates band corresponding to ATXN3 from previous detection. (c) Cell lysates from HeLa cells expressing dox‐inducible ^10xHis^ATXN3^HA^ were incubated with purified, recombinant GST‐tagged Atg8 proteins and binding analyzed by immunoblotting with the indicated antibodies. (d) Schematic illustration of GFP‐ATXN3 constructs used in (e). (e) Cell lysates from HeLa cells transfected with the indicated constructs were incubated with blocked empty agarose beads (e.b.) or recombinant GABARAP immobilized on agarose beads. Binding was analyzed with immunoblotting using the indicated antibodies

In light of its promiscuous interaction with various ubiquitin‐related proteins (Burnett, Li, & Pittman, 2003; Ferro et al., 2007; Pfeiffer et al., 2017), we wondered whether ATXN3 binds the autophagy receptors LC3 and GABARAP, which belong to the Atg8 family of ubiquitin‐like modifiers (Schaaf, Keulers, Vooijs, & Rouschop, 2016). To this end, we incubated recombinant ATXN3 with LC3A and GABARAP agarose beads, which revealed a robust interaction between ATXN3 and GABARAP, while binding of ATXN3 to LC3A was not observed (Figure 4a). Similar to recombinant ATXN3, we found that ectopically expressed ATXN3 with N‐terminal histidine (10xHis) and C‐terminal hemagglutinin (HA) epitope tags (^10xHis^ATXN3^HA^) as well as endogenous ATXN3 binds GABARAP. Additionally, interaction with LC3A was also detected, albeit much less pronounced (Figure 4b). As an internal control, we validated that p62, a known Atg8‐interacting protein, was precipitated by the beads. The specificity of the ATXN3 signal was confirmed, as induction of ^10xHis^ATXN3^HA^ increased the amounts of ATXN3 in the GABARAP precipitates, while siRNA‐mediated depletion of ATXN3 resulted in a loss of the ATXN3 signal (Figure S5).

As there are six Atg8 family members in mammalian cells, we next sought to characterize the specificity of ATXN3 for these proteins further. Lysates of HeLa ^10xHis^ATXN3^HA^ were incubated with GST‐LC3A, GST‐LC3B, GST‐LC3C, GST‐GABARAP, GST‐GABARAPL1, or GST‐GABARAPL2 immobilized on glutathione beads, and precipitates were analyzed for ^10xHis^ATXN3^HA^. This approach confirmed that ^10xHis^ATXN3^HA^ precipitated with GST‐GABARAP and to a lesser extent with GST‐LC3A. Additionally, we detected strong binding of GST‐LC3C to ^10xHis^ATXN3^HA^ (Figure 4c). To define the domain in ATXN3 that is required for GABARAP binding, we generated a series of green fluorescent protein (GFP)‐tagged fragments of ATXN3 that were ectopically expressed in HeLa cells (Figure 4d). Incubation of the cell lysates with GABARAP‐coated beads revealed that the N‐terminal half of ATXN3, comprising the catalytic Josephin domain, interacted with GABARAP (Figure 4e). Further deconvolution showed that the interaction between the C‐terminal part of the Josephin domain and GABARAP was most pronounced with weaker binding of GABARAP to the N‐terminal part of the Josephin domain (Figure 4e). These results suggest that ATXN3 interacts with GABARAP, and likely LC3C, through multiple binding sites in its Josephin domain.

### 2.5 The Josephin domain contains two conserved LIR motifs

**Figure 5:**
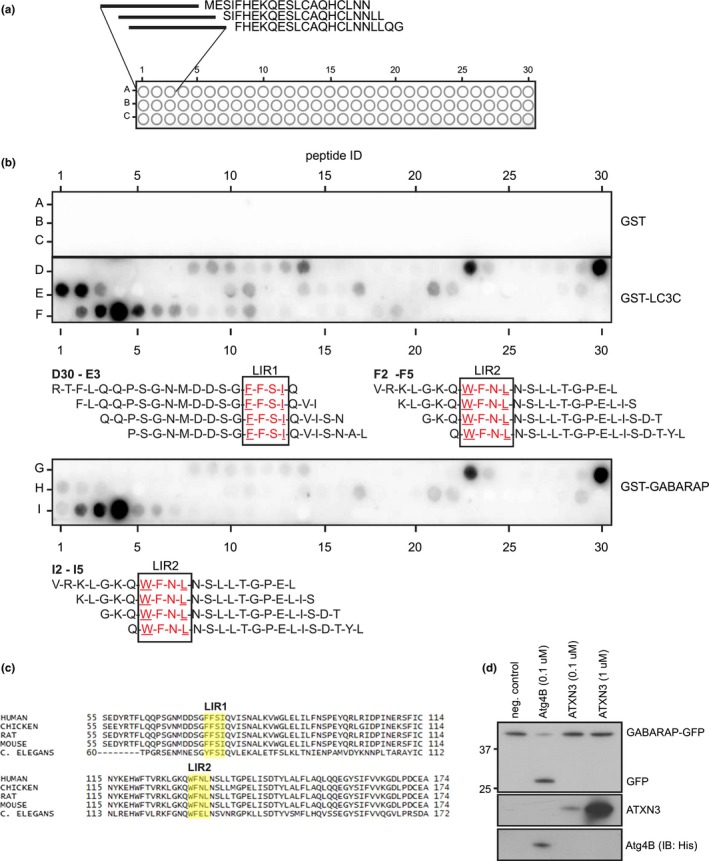
The ATXN3 Josephin domain contains two conserved LIR motifs. (a) Schematic illustration of peptide spot assay to identify LIR motifs in the ATXN3 Josephin domain. The N‐terminal sequence of ATXN3 (1–182) was synthesized as 20‐mer peptides and spotted on cellulose membranes. (b) Binding of GST‐tagged fusions of GABARAP and LC3C to the spotted ATXN3 Josephin peptides was analyzed by immunoblotting. (c) Conservation of the identified LIR1 and LIR2 across species. (d) Catalytic activity of recombinant ATXN3 toward recombinant pre‐GABARAP‐GFP was analyzed in vitro by incubation of 250 ng pre‐GABARAP‐GFP with the indicated amounts of ATXN3 for 16 hr at 37°C. Recombinant His_6_‐Atg4B was used as control

In silico analysis of the ATXN3 sequence (NP_004984.2), using iLIR (Kalvari et al., 2014), revealed the presence of six putative LIRs ([W/F/Y]‐x‐x‐[L/V/I]) with variable PSSM scores (Figure S6A,B). All putative LIRs were located in the Josephin domain, whereas no candidate LIRs were identified in the C‐terminal part of ATXN3. To identify the LIR(s) responsible for the GABARAP/LC3C interaction, we synthesized and spotted the N‐terminal sequence of ATXN3 (1–182), encompassing the Josephin domain, as 20‐mer peptides on cellulose membranes, and analyzed binding of GST‐tagged fusions of GABARAP and LC3C to the immobilized peptides (Figure 5a,b). LC3C bound to a LIR sequence located in the central part of the Josephin domain (FFSI, aa 74–77, LIR1), while both GABARAP and LC3C bound to peptides containing a LIR sequence located more toward the C‐terminal part of the Josephin domain (WFNL, aa130‐133, LIR2) (Figure 5b, Figure S6B). This observation may also provide an explanation for the observed binding of both halves of the Josephin domain to GABARAP as LIR1 and LIR2 are located in the N‐terminal and C‐terminal fragments of the Josephin domain, respectively. Both LIRs are highly conserved in ATXN3 orthologues among various species, suggesting a conserved function for these motifs (Figure 5c). We assessed the catalytic activity of ATXN3 mutants in which the critical residues in LIR1 or LIR2 or both had been mutated toward K63‐linked hexa‐ubiquitin chains in vitro and found that both mutations compromised the deubiquitylation activity of ATXN3 (Figure S6C). For the LIR1 mutant, this result was expected as this LIR motif shares a critical residue with the ubiquitin‐binding site 1 (UbS1; Ile77Glu78) in the ATXN3 Josephin domain. This site has recently been shown to be crucial for ATXN3 activity as it is required to position the ubiquitin C‐terminus for cleavage (Nicastro et al., 2010). LIR2 is located directly adjacent to Asp134, an essential residue of the catalytic triad of ATXN3, which may explain why mutating LIR2 renders ATXN3 inactive. Since the expression of an ATXN3 LIR2 mutant in mammalian cells and protein purification from bacteria turned out to be problematic with low expression and yields, we suspect that these mutations may also impair folding of the catalytic domain. Due to the lack of DUB activity of these mutants combined with the low expression levels, we have been unable to address the significance of these LIRs for the functional role of ATXN3 in autophagy.

The specific interaction of the catalytic domain of ATXN3 with GABARAP and LC3C raised the question whether ATXN3 displays protease activity toward these ubiquitin‐like proteins. While the authentic protease of GABARAP, Atg4B (Tanida et al., 2004), readily cleaved a recombinant GABARAP‐GFP fusion protein, ATXN3 did not display any activity toward this fusion protein (Figure 5d).

### 2.6 ATXN3 localizes to autophagosomes

**Figure 6:**
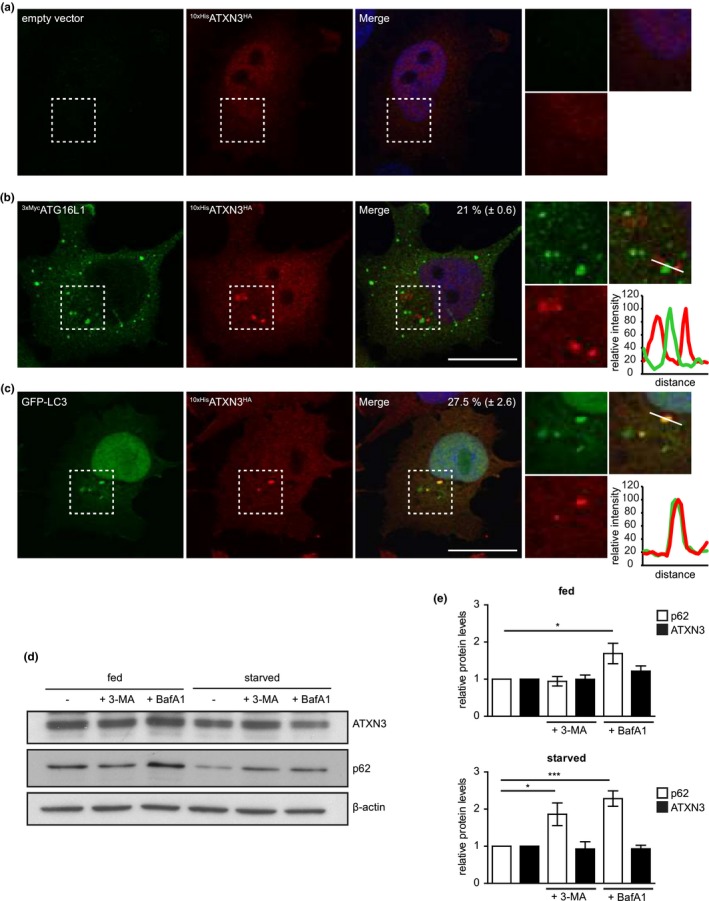
ATXN3 localizes at autophagosomes. (a, b) HeLa cells expressing dox‐inducible ^10xHis^ATXN3^HA^ were transfected with empty vector (a) or ^3xMyc^ATG16L1 (b) and ^10xHis^ATXN3^HA^ expression induced for 24 hr. Cells were starved for 2 hr, fixed, and immunolabeled using antibodies against HA‐tag and Myc‐tag. Line scan at site of ^3xMyc^ATG16L1 accumulation to visualize the spatial distribution of the proteins. The percentage of ^3xMyc^ATG16L1 puncta that were associated with ATXN3 was quantified. Data are presented as mean ± *SEM* of four independent experiments. (c) HeLa cells expressing dox‐inducible ^10xHis^ATXN3^HA^ were transfected with GFP‐LC3 and ^10xHis^ATXN3^HA^ expression induced for 24 hr. Cells were starved for 2 hr, fixed, and immunolabeled using antibodies against HA. Line scan at site of GFP‐LC3 accumulation to visualize the spatial distribution of the proteins. The percentage of GFP‐LC3 puncta that co‐localized with ATXN3 was quantified. Data are presented as mean ± *SEM* of three independent experiments (d) HeLa cells were treated as indicated for 4 hr and cell lysates analyzed by immunoblotting using the indicated antibodies. (e) Band intensities were quantified and normalized to β‐actin and to the respective control treatment. Data are presented as mean ± *SD* of three independent experiments. *p ≤ .05, **p ≤ .01, ***p ≤ .001 (one‐way ANOVA)

Based on the interactions identified above, we wondered whether ATXN3 localizes to autophagosomes. To this end, we analyzed the distribution of ATXN3 in HeLa cells under control conditions, when basal autophagy is active, and under nutrient starvation to stimulate autophagy. Epitope‐tagged ^10xHis^ATXN3^HA^ showed a diffuse staining in both the cytosol and nucleus, which remained unchanged during starvation (Figure 6a, Figure S7A). Next, we analyzed the localization of ^10xHis^ATXN3^HA^ in cells that overexpressed 3xMyc‐tagged ATG16L1 (^3xMyc^ATG16L1) as this is known to induce the formation of pre‐autophagosomal structures and stall their progression (Polson et al., 2010). As expected, this resulted in an increase in WIPI2‐positive puncta, a marker for early autophagosomes (Polson et al., 2010), which co‐localized with the ectopically overexpressed ^3xMyc^ATG16L1 (Figure S8). Notably, ATG6L1 overexpression resulted in the appearance of distinct ATXN3 puncta, which typically localized in close proximity to ATG16L1 structures under basal and starved conditions (Figure 6b, Figure S7B). Overexpression of GFP‐tagged LC3 also resulted in ATXN3 re‐localization to distinct puncta, where ATXN3 co‐localized with the GFP‐LC3 foci, consistent with translocation of ATXN3 to autophagosomes (Figure 6c).

The observation that ATXN3 localizes to autophagosomes upon ATG16L1 overexpression suggests that ATXN3 transiently interacts with early autophagosomal structures but does not progress through the autophagic pathway. This also implies that ATXN3 is not targeted for lysosomal degradation unlike other LC3/GABARAP‐interacting proteins, such as p62 (Pankiv et al., 2007). Consistent with this notion, inhibition of autophagy by 3‐methyladenine (3‐MA) or bafilomycin A1 in nutrient‐starved cells did not result in an increase in the steady‐state levels of ATXN3, whereas p62 was stabilized (Figure 6d,e). We conclude that ATXN3 is an LC3C/GABARAP‐interacting protein that is recruited to autophagosomes in a transient fashion, where it regulates their formation and stimulates the autophagic flux.

## 3 DISCUSSION

In this study, we identified ATXN3 as a GABARAP/LC3C‐interacting DUB that localizes to autophagosomes and revealed a novel regulatory function in autophagy. Surprisingly, we found that nematodes deficient for ATX‐3 and CDC‐48, which have an approximate 50% expansion of their lifespan, are hypersensitive to starvation conditions, resulting in reduced survival of food‐deprived mutant larvae. The starvation response required the catalytic activity of ATX‐3, while CDC‐48 deletion did not decrease viability of the worms in response to nutrient starvation. Since longevity is usually associated with increased autophagy (Leidal et al., 2018), it is striking that the extended lifespan of the *cdc‐48.1; atx‐3* double mutant worms occurs at the expense of the ability to cope with prolonged starvation. Interestingly, *atx‐3* mutant worms do not show increased sensitivity toward proteotoxic insults such as high temperature, oxidative stress, or bortezomib treatment (Kuhlbrodt et al., 2011; Rodrigues et al., 2007). Thus, the specific decrease in the survival of *atx‐3* mutant worms under starvation stress is indicative of a specialized role of ATX‐3 in the fine‐tuning of autophagy to survive unfavorable environmental conditions.

Our results are in striking contrast to the recent observation by Ashkenazi and coworkers, who identified the autophagy‐regulating factor beclin‐1 as a target for ATXN3 deubiquitylating activity and demonstrated polyQ‐mediated binding between beclin‐1/ATXN3. Stabilization of beclin‐1 by ATXN3 was proposed to explain the stimulatory role of ATXN3 on LC3 lipidation (Ashkenazi et al., 2017). Even though our data also argue for a stimulatory role of ATXN3 in autophagy, within the shorter time frame of ATXN3 depletion that we employed in this study, neither beclin‐1 levels nor LGG‐1/LC3 lipidation was reduced upon ATX‐3 or ATXN3 depletion. Moreover, the starvation sensitivity phenotype of *atx‐3* deletion worms was aggravated by crippling autophagy (*bec‐1* or *unc‐51* depletion), suggesting a conserved beclin‐1‐independent role in autophagy and degradation of long‐lived proteins. It is also noteworthy that *C. elegans* ATX‐3 lacks the polyQ tract present in the human protein (Rodrigues et al., 2007), which shows that ATX‐3 is able to stimulate autophagy independent of a polyQ stretch and strongly argues in favor of a function different from the polyQ‐dependent regulation of beclin‐1.

ATXN3 joined the arsenal of neurodegeneration‐associated proteins that have been found to promote various events in the autophagy–lysosome pathway (Deng et al., 2017; Wong & Cuervo, 2010). The shared commitment of these proteins to autophagy brings up important questions about the underlying pathophysiological mechanisms of these diseases, which are characterized by the accumulation of aggregation‐prone proteins. It is plausible that defects in protein degradation may contribute to disease manifestation. Interestingly, genetic tempering with autophagy in mice gives rise to neurodegenerative disorders with the presence of ubiquitin‐positive protein inclusions that are hallmarks of these diseases (Hara et al., 2006; Komatsu et al., 2006). With respect to ATXN3, the recent identification of Huntingtin, the polyQ protein responsible for Huntington's disease, as a substrate adaptor for autophagy, appears to be most relevant (Rui et al., 2015). A feature that ATXN3 shares with Huntingtin and other cargo receptors is the binding of both GABARAP/LC3 and ubiquitin conjugates. However, our data do not support a classical role of ATXN3 in substrate delivery since autophagy receptors typically join their substrates to the lysosomes and are degraded in the process. In contrast, we did not detect lysosomal degradation of ATXN3. Hence, the identified LIR motifs might be required for the recruitment of ATXN3 to autophagosomes in a more temporally controlled manner. Such a mechanism would not be unprecedented since similar targeting functions of LIR motifs have been described for other effector proteins in the autophagy pathway, including ULK1, ATG4B, and mitogen‐activated protein kinase 15 (MAPK15) (Alemu et al., 2012; Colecchia et al., 2012; Skytte Rasmussen et al., 2017).

The functional significance of the two identified LIRs will require further exploration before definite conclusions can be drawn. Analysis of the published structures of the Josephin domain indicates that the LIR1 motif is partly exposed but will have to undergo a minor conformational change to align properly (Mao et al., 2005; Nicastro et al., 2005). Due to its proximity to the UbS1 site (Nicastro et al., 2010), occupancy of LIR1 with LC3C will likely hinder binding of ubiquitin and interfere with its DUB activity. The LIR2 motif, on the other hand, is buried within the Josephin domain and is of structural importance for the integrity of this domain. Exposure of the LIR2 motif to GABARAP/LC3C would therefore require a conformational change in the Josephin domain. While conformational changes upon substrate engagement are not uncommon in DUBs (Hu et al., 2002; Johnston, Riddle, Cohen, & Hill, 1999), a quite dramatic structural reorganization of the catalytic domain would be required to surface expose this LIR motif, making it unlikely, though not impossible, that it contributes to the interaction between LC3C/GABARAP and ATXN3. The fact that binding of LC3C/GABARAP to the LIR(s) may inhibit the catalytic activity of ATXN3, while at the same time the catalytic activity is required for its role in autophagy, is intriguing. One possibility is that there is a relay mechanism in play where ATXN3 is first recruited to autophagosomes by binding LC3C/GABARAP followed by the replacement of GABARAP/LC3C for a ubiquitylated substrate. This model could also explain the transient nature of its interaction with autophagosomes. Alternatively, the catalytic activity of ATXN3 may be required for processing substrates other than ubiquitin chains. While we did not detect any activity toward an artificial GABARAP precursor, this does not yet exclude the possibility that ATXN3 may process conjugates of GABARAP/LC3C or other Atg8 members.

Counterintuitively, ATXN3 depletion increases the number of LC3 puncta without an accompanying increment in lipidated LC3. It is plausible that local inhibition of the formation of autophagosomes by ATXN3 may ensure that the available pool of lipidated LC3 is not diluted among an excessively large number of autophagosomes, which may otherwise hinder efficient autophagic clearance. This model would explain why ATXN3 only localizes to a subpopulation of ATG16L1‐positive structures and why ATXN3 is not degraded in the process despite its co‐localization with markers for autophagosomes. Thus, we propose that ATXN3 may localize to a subpopulation of ATG16L1 structures that will not further progress to the formation of autophagosomes due to the suppressive activity of ATXN3. Although speculative, these models provide us with a framework for further exploration of the molecular role of ATXN3 in autophagy. Together, we conclude that ATXN3 stimulates autophagy and thereby optimizes the cellular response to nutrient starvation and proteotoxic stress, two conditions that rely heavily on autophagy.

## 4 EXPERIMENTAL PROCEDURES

### 4.1 Starvation of worms

Worms were maintained at 20°C on NGM plates seeded with *Escherichia coli* (OP50). Gravid adult worms were washed off plates and treated with alkaline bleach (7.7 ml H_2_O + 2 ml 10% bleach + 0.3 ml 10 M NaOH) to isolate embryos. The isolated embryos were hatched in M9 buffer at 20°C without food overnight (ca. 10–50 eggs/μl). The arrested first‐stage larvae (L1) were incubated in M9 buffer, rotating at 20°C for the indicated number of days. To monitor starvation survival as the recovery rate upon refeeding, aliquots from each sample were taken every 1–3 days and plated on 3.5 cm NGM plates seeded with *E. coli* (OP50) for recovery. After 2 days of recovery at 20°C, surviving worms that had resumed growth and developed past the L1 larval stage were counted and removed. Plates were inspected after an additional 24 hr to count worms that developed slower and were missed in the first round of counting. The recovery rate observed on the first day of starvation (24 hr after egg isolation and incubation in M9 buffer) was set to 100% and recovery rates of the following days normalized to this.

### 4.2 Motility assay

Worms were grown on *E. coli* (OP50) bacteria at 20°C until day 1 adulthood (72 hr after L1 stage). Worms were transferred to a drop of M9 buffer, and after 30 s of adaptation, the number of body bends of *n* = 20 worms was counted for 30 s. A body bend was defined as change in direction of the bend at the mid‐body.

### 4.3 Long‐lived protein degradation

Reverse transfection of U2OS cells was done by dilution of siRNA oligonucleotides and Lipofectamine 2000 in DMEM + GlutaMAX. Cells were added at a density of 8.8 × 10^4^ cells/ml in a final volume of 400 μl (final siRNA concentration: 40 nM). Cells were labeled by the addition of 0.125 μCi/ml L‐[^14^C] valine to the medium for 24 hr, followed by two washes and a 16‐hr chase in medium containing 10 mM nonradioactive l‐valine, to allow degradation of short‐lived proteins. Subsequently, cells were washed and subjected to the indicated treatments in medium containing 10 mM nonradioactive l‐valine for 4 hr. Radioactivity in the acid‐soluble and acid‐insoluble fractions was measured using Ultima Gold LSC cocktail (Perkin Elmer) and a liquid scintillation counter (Tri‐Carb 3100T Perkin Elmer).

### 4.4 Peptide scan

The N‐terminal sequence of human ATXN3 (aa 1–182; NP_004984.2) was synthesized as 20‐mer peptides with two amino acid offsets on cellulose membranes using a MultiPep automated peptide synthesizer (INTAVIS Bioanalytical Instruments) as described (Knaevelsrud et al., 2013). Peptide arrays were blocked in 1% casein in PBS‐T for 1 hr at RT. Overlays were done using 1 μg/ml GST‐fusion proteins or GST in 1% casein PBS‐T overnight at 4°C. Membranes were washed three times in PBS‐T and bound proteins detected by immunoblotting with anti‐GST‐HRP.

See Supporting Information Experimental Procedures for all additional experimental procedures.

## CONFLICT OF INTEREST

No competing interests declared.

## AUTHOR CONTRIBUTIONS

LKH, EK, TH, and NPD designed the research; LKH, EK, RM, AHL, CB, CB, AP, and MEG performed the experiments; LKH, EK, RM, AHL, CB, FAS, AS, TH, and NPD analyzed the data; and LKH, EK, TH, and NPD wrote the manuscript.

## Data Availability

The data that support the findings of this study are available from the corresponding author upon reasonable request.
