# Supplementary material for: The Machado–Joseph disease deubiquitylase ataxin‐3 interacts with LC3C/GABARAP and promotes autophagy
Source: Aging Cell. 2019 Oct 17;19(1):e13051. doi: 10.1111/acel.13051 (PMC6974715; doi:10.1111/acel.13051)
Supplement: Supplementary file 1 [file ACEL-19-e13051-s001.pdf]

Supporting Information Figure Legends

Supporting Information Figure S1: L1 larvae of *atx-3* deletion strain exhibit phenotypes that are consistent with reduced autophagy activity. (A) Survival of *atx-3(gk193)* mutant worms upon RNAi-mediated reduction of *unc-51* expression and starvation. Downregulation of *unc-51* decreases survival of *atx-3* mutant L1 larvae during starvation. Graphs show mean survival ± SEM of L1 larvae upon re-feeding. Data are derived from eight biological replicates ($n \geq 250$). **(B)** Expression of the proteotoxic polyglutamine species in *atx-3* mutant worms. Representative fluorescent images of *atx-3(gk193)* mutant and wild-type worms expressing Q40::YFP aggregation-prone protein in body-wall muscle cells on day 1 adulthood (24h after L4 stage). Scale bar: 80 μm.

Supporting Information Figure S2: ATXN3 depletion in mammalian cells results in autophagy defects without affecting the steady-state levels of autophagy proteins. (A) Western blot analysis of wild-type (WT) and ATXN3 deletion (KO) mouse embryonic fibroblasts (MEFs). **(B)** Quantification of endogenous LC3B puncta in WT or ATXN3 KO MEFs shown in Figure 2F. Cells were treated as indicated for 4h. * $p \leq 0.05$, ** $p \leq 0.01$, *** $p \leq 0.001$ (Kruskal-Wallis). **(C)** Representative micrographs of HOS GFP-LC3 cells depleted of ATXN3 using two different siRNAs (ATXN3si #1, ATXN3si #2) and starved in the presence of Wortmannin for 4h. Cells were imaged live using an automated widefield microscope. Scale bar: 20 μm. **(D)** HeLa cells transfected using CNTRLsi, ATXN3si #1 or ATXN3si #2 were treated as indicated for 4h and the steady-state levels of autophagy proteins analysed by immunoblotting using the indicated antibodies. **(E)** Similar to (D) in U2OS cells.

Supporting Information Figure S3: Flow cytometry analysis of autophagic flux and relative cellular mRFP- and GFP-intensities in U2OS mRFP-GFP-LC3 cells upon ATXN3 depletion. (A) U2OS mRFP-GFP-LC3 cells were transfected using CNTRLsi, ATXN3si #1 or ATXN3si #2, treated as indicated for 4h, washed briefly in 0.05 % saponin and analysed by flow cytometry. Autophagic flux was determined as the ratio of mean mRFP- and GFP-fluorescence and normalized to CNTRLsi-treated cells, starved in the presence of BafA1. Data are presented as mean ± SD of four independent experiments. **(B, C)** Mean GFP-fluorescence **(B)** and mRFP-fluorescence **(C)** of cells in (A). Data were normalized to CNTRLsi-treated, starved cells. Data are presented as mean ± SD of four independent experiments. **(D, E)** Representative histograms of GFP- **(D)** and mRFP-fluorescence **(E)** from one of four independent experiments are shown.

Supporting Information Figure S4: Knockdown efficiency of endogenous ATXN3 and transient expression of mCherry-ATXN3 in HOS GFP-LC3. Cells were transfected with ATXN3si #2 for 24h prior to transfection with mCherry (mCh.), mCherry-ATXN3 WT (WT) or mCherry-ATXN3 C14A (C14A) for 24h and analysed by immunoblotting with the indicated antibodies.

Supporting Information Figure S5: Interaction of ATXN3 with GABARAP is specific. Cell lysates of HeLa cells expressing dox-inducible ^{10xHis}ATXN3^{HA} transfected with CNTRLsi or ATXN3si #2 were incubated with blocked agarose beads (e.b.) or recombinant GABARAP immobilized on agarose beads. Binding of endogenous ATXN3 and ^{10xHis}ATXN3^{HA} was analysed by immunoblotting with the indicated antibodies.

Supporting Information Figure S6: Identification of LIR motifs in ATXN3 Josephin domain and analysis of catalytic activity ATXN3 LIR mutants. (A) *In silico* analysis of the ATXN3 sequence using iLIR revealed six putative LIR motifs in the ATXN3 Josephin domain. **(B)** Schematic illustration of six putative LIRs across ATXN3 Josephin domain. **(C)** Catalytic activity of recombinant ATXN3 LIR mutants (mutation of position one and four in canonical WxxL-LIR in ATXN3 LIR1 or LIR2 or both) was analysed *in vitro* using hexa-ubiquitin (K63-linked) as substrate. Reactions were performed for 16h and analysed by immunoblotting with the indicated antibodies. Wild-type ATXN3 was used as control.

Supporting Information Figure S7: ^{10xHis}ATXN3^{HA} localizes in proximity to ^{3xMyc}ATG16L1-induced stalled phagophores under fed and starved conditions. (A) HeLa cells expressing dox-inducible ^{10xHis}ATXN3^{HA} transfected with empty vector were treated as indicated for 2h, fixed and immunolabelled using antibodies against Myc-tag and HA-tag. **(B)** HeLa cells expressing dox-inducible ^{10xHis}ATXN3^{HA} transfected with ^{3xMyc}ATG16L1. Cells were treated as indicated for 2h, fixed and immunolabelled using antibodies against Myc-tag and HA-tag.

Supporting Information Figure S8: ^{3xMyc}ATG16L1-induced stalled phagophores are positive for WIPI2. HeLa cells expressing dox-inducible ^{10xHis}ATXN3^{HA} transfected with empty vector or ^{3xMyc}ATG16L1. Cells were starved for 2h, fixed and immunolabelled using antibodies against Myc-tag and WIPI2. Scale bar: 20 μm.

Supporting Information Figure S1

A

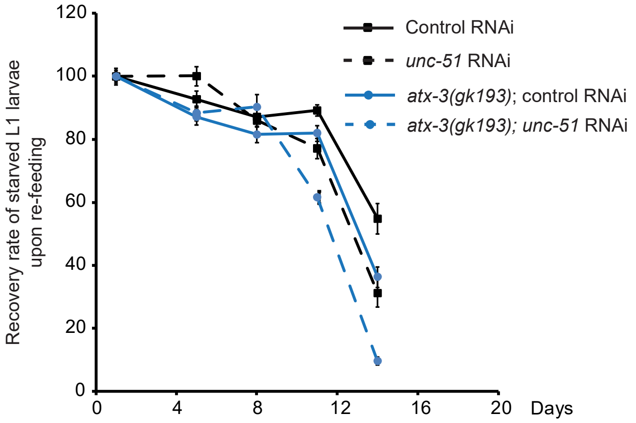

B

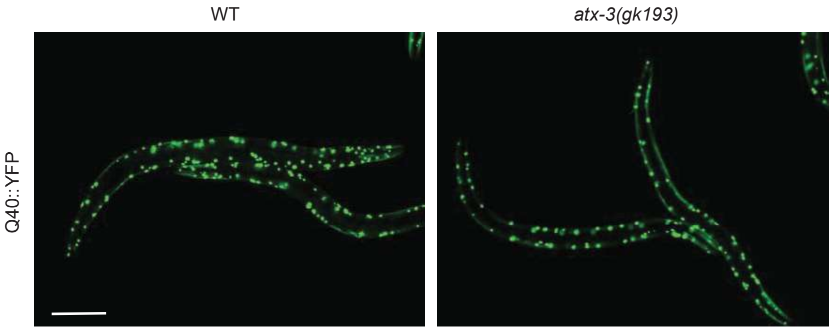

Supporting Information Figure S2

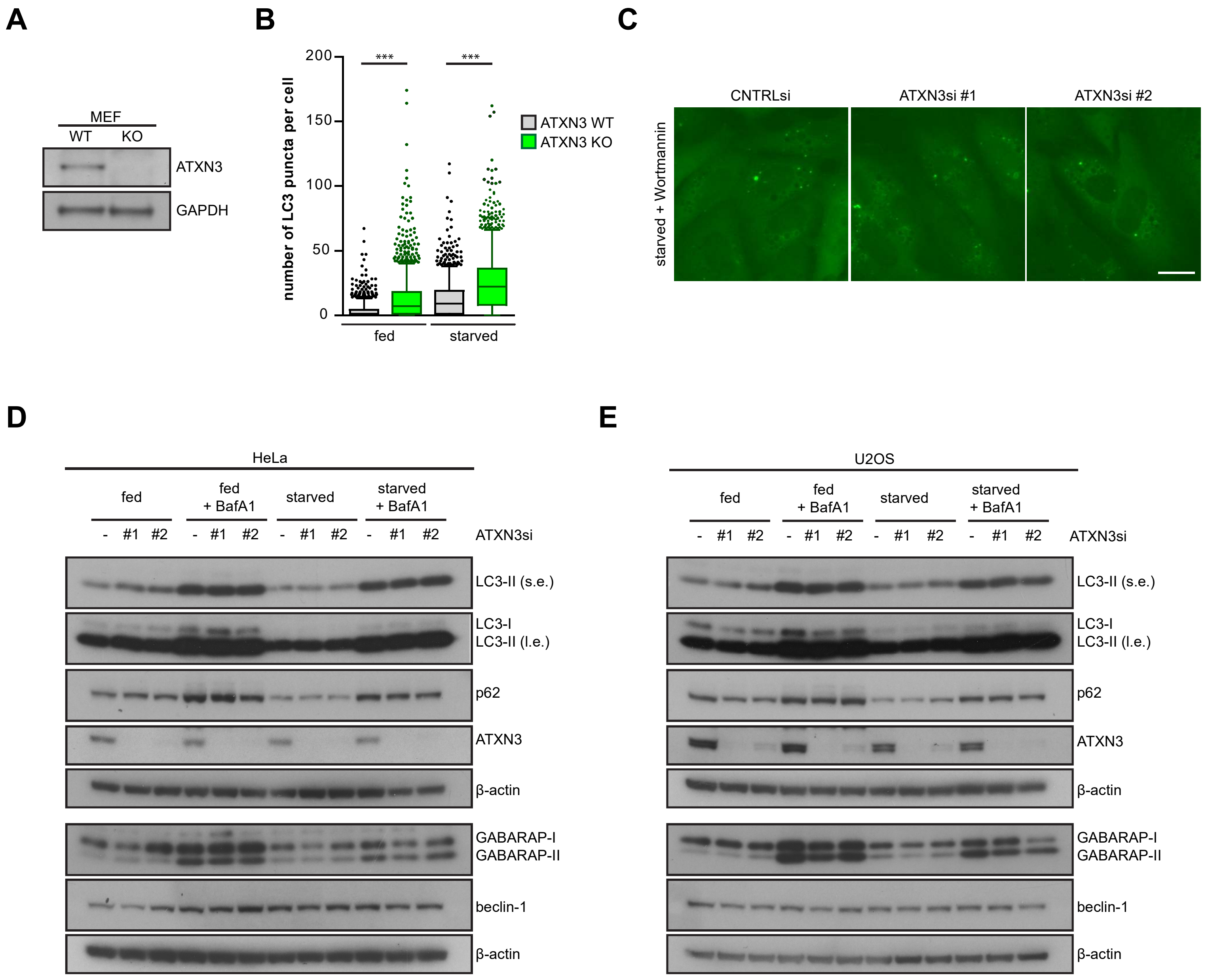

Supporting Information Figure S3

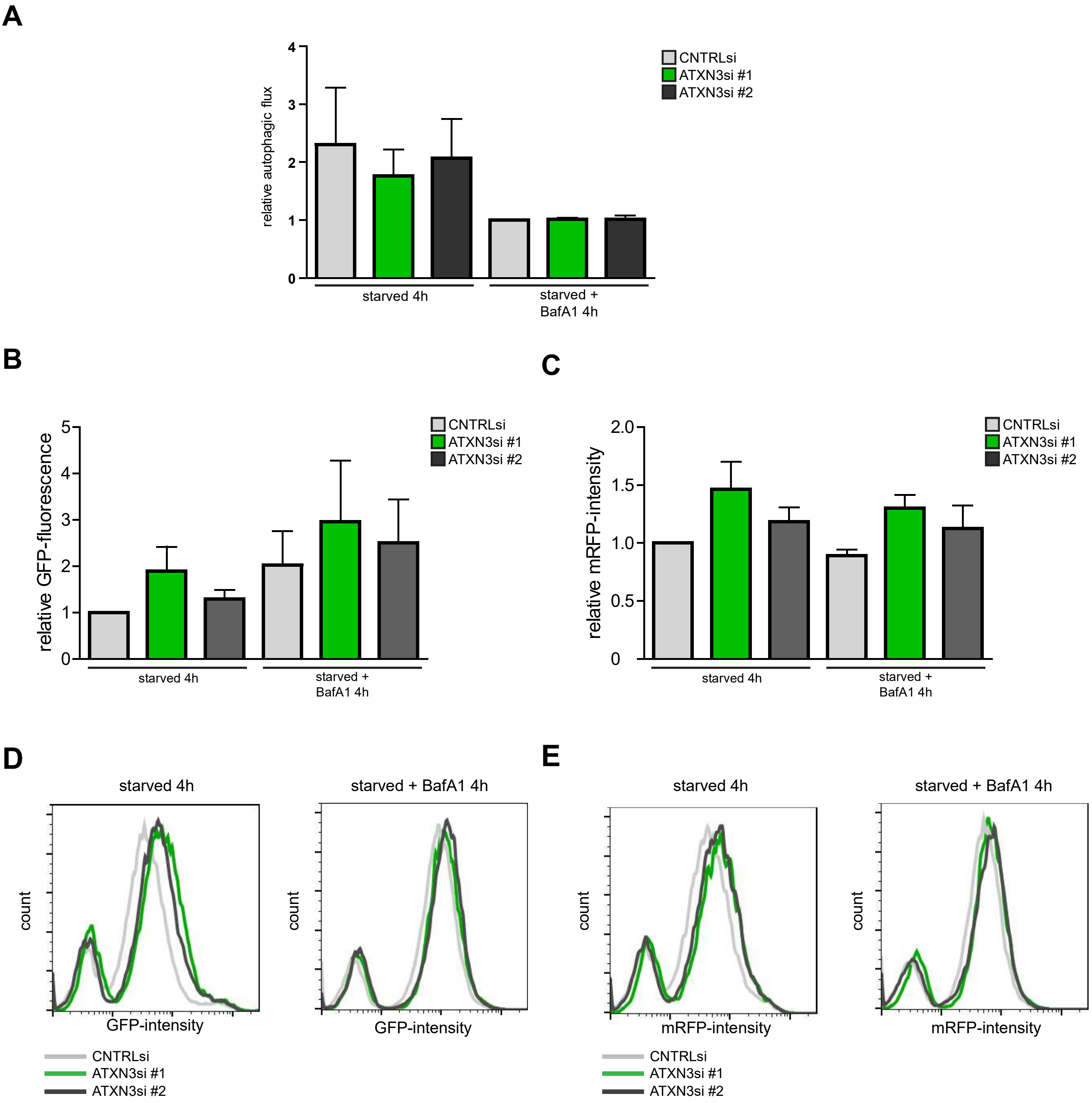

Supporting Information Figure S4

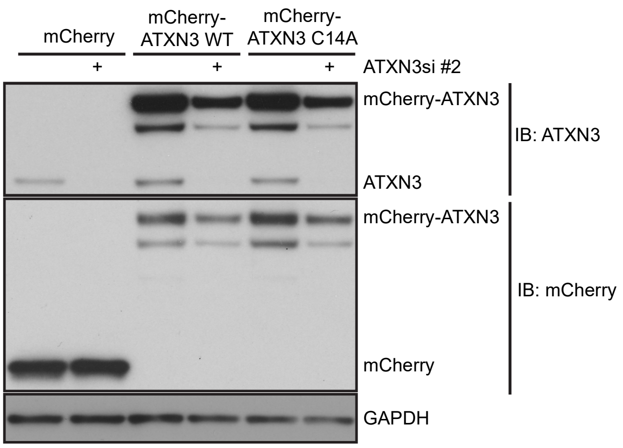

Supporting Information Figure S5

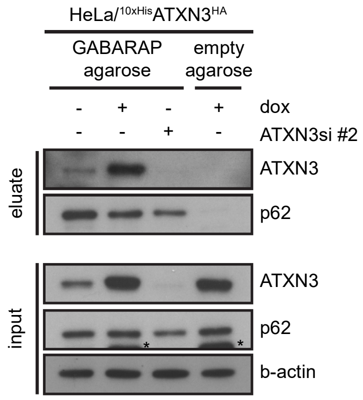

Supporting Information Figure S6

A

putative LIR motifs in ATXN3 identified using iLIR

Motif	Start	End	LIR sequence	PSSM score	similar LIRs
WxxL	26	31	EY**F**SP**V**	4 (1.4e+00)	-
WxxL	72	77	SG**F**SS**I**	8 (3.9e-01)	-
WxxL	97	102	PE**Y**QR**L**	11 (1.5e-01)	-
WxxI	118	123	EH**W**TF**V**	12 (1.1e-01)	-
WxxL	128	133	KQ**W**FN**L**	10 (2.0e-01)	-
xLIR	145	150	DT**Y**LA**L**	14 (5.7e-02)	-

(http://repeat.biol.ucy.ac.cy/cgi-bin/iLIR/iLIR_cgi)

B

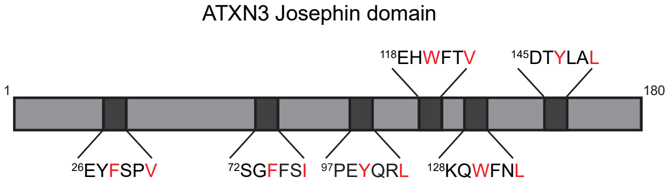

C

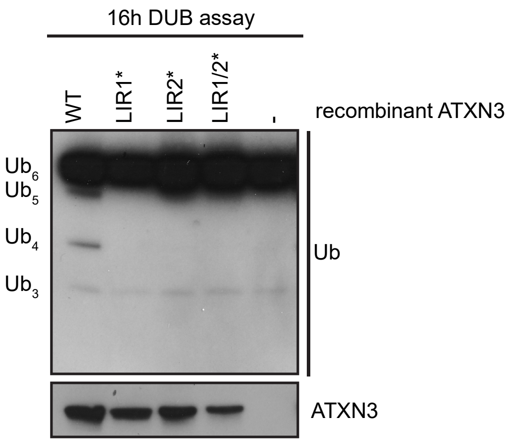

Supporting Information Figure S7

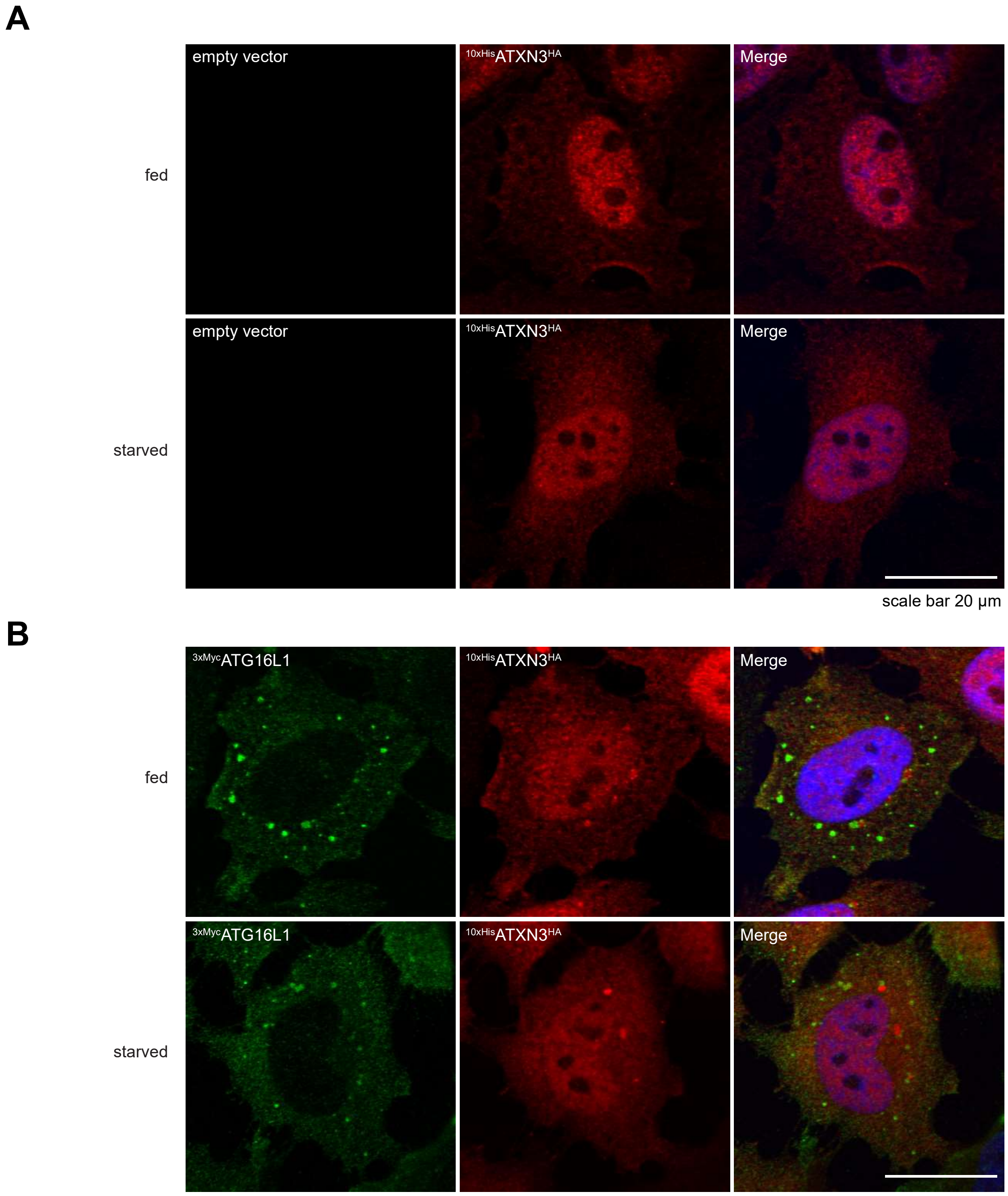

Supporting Information Figure S8

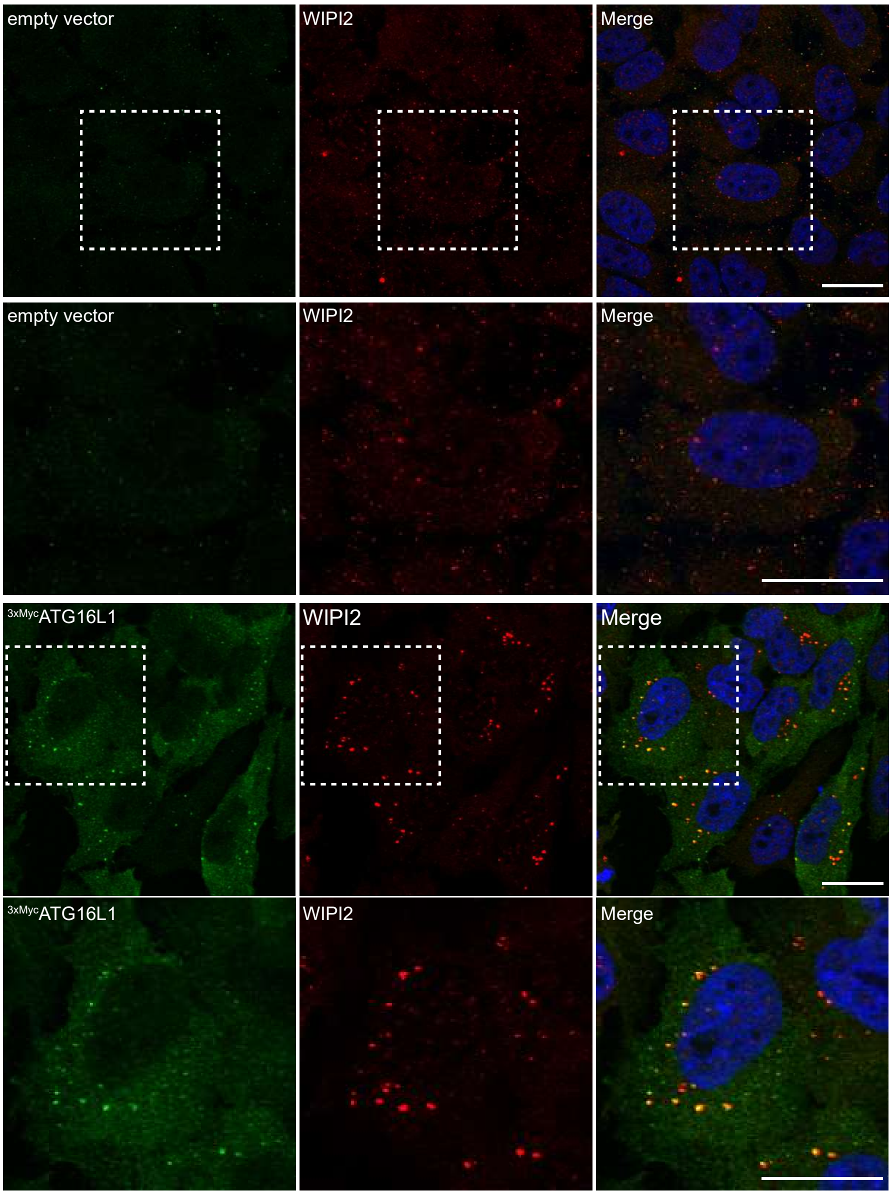

Supporting Information Table S1: Statistical analysis for Figure 1A

		Day				
		1	6	8	11	14
N2	Average	92,093	88,682	95,000	75,734	39,331
	SEM	3,646	3,599	5,270	5,242	5,064
atx-3(gk193)	Average	96,519	91,099	92,892	60,329	18,302
	SEM	0,319	3,164	2,105	3,730	1,925
	t-test vs N2					**
		0,261128	0,627577	0,736614	0,072071	0,006421
cdc-48.1(tm544); atx-3(gk193)	Average	96,612	91,981	83,450	42,728	5,472
	SEM	1,573	3,033	6,467	6,631	1,541
	t-test vs N2				**	***
		0,288111	0,503236	0,203584	0,004513	0,000210
cdc-48.1(tm544)	Average	93,067	93,331	97,500	81,922	44,782
	SEM	4,935	2,285	2,500	6,484	4,070
	t-test vs N2					
		0,877800	0,307210	0,679510	0,479170	0,425830

Average - Average survival for the designated time point (%)
SEM - Standard error of the mean
$n = 5$
* $p \leq 0.05$, ** $p \leq 0.01$, *** $p \leq 0.001$

Supporting Information Table S2: Statistical analysis for Figure 1B

		Day						
		1	4	5	6	9	12	15
N2	Average	92,707	94,902	96,292	95,405	90,845	51,843	12,963
	SEM	1,799	1,411	1,846	1,716	3,214	2,721	1,499
atx-3(gk193)	Average	89,647	90,385	89,570	78,953	76,887	34,696	3,114
	SEM	2,497	2,417	2,206	2,043	2,053	2,523	0,986
	t-test vs N2			*	***	**	***	***
		0,368158	0,153317	0,046326	0,000049	0,004114	0,000703	0,000152
atx-3(tm1689)	Average	86,541	86,184	85,882	84,550	62,822	36,740	0,899
	SEM	2,208	2,085	3,014	2,538	2,541	3,600	0,500
	t-test vs N2		**	*	**	***	**	***
		0,062303	0,005933	0,015486	0,005120	0,000017	0,007368	0,000005

Average - Average survival for the designated time point (%)
SEM - Standard error of the mean
n = 8
* $p \leq 0.05$, ** $p \leq 0.01$, *** $p \leq 0.001$

Supporting Information Table S3: Statistical analysis for Figure 1C

		Day						
		1	3	6	8	10	13	15
N2	Average	94,159	95,202	91,486	95,818	92,542	74,328	42,375
	SEM	1,458	1,114	2,025	1,533	1,969	2,048	2,606
gk193 atx3::gfp	Average	95,953	89,649	92,827	86,906	88,442	67,652	38,826
	SEM	0,735	2,575	0,818	1,528	1,297	5,866	1,849
	t-test vs N2	0,290503	0,067778	0,549272	** 0,001047	0,103940	0,300804	0,285266
gk193 atx3_C20S::gfp	Average	95,488	90,930	91,288	89,465	84,533	47,125	22,910
	SEM	1,019	2,206	1,913	1,621	1,792	2,754	2,537
	t-test vs N2	0,467447	0,105952	0,944317	0,012919	** 0,009394	*** 0,000002	*** 0,000102
	t-test vs *gk193 atx3::gfp*	0,716703	0,711144	0,471758	0,269941	0,099044	** 0,006849	*** 0,000171

Average - Average survival for the designated time point (%)
SEM - Standard error of the mean
n = 8
* $p \leq 0.05$, ** $p \leq 0.01$, *** $p \leq 0.001$

Supporting Information Table S4: Statistical analysis for Figure 1D

		Day					
		1	2	4	8	12	14
N2_control RNAi	Average	96,188	95,947	92,785	82,100	22,155	1,666
	SEM	1,949	1,113	1,640	2,312	1,951	0,433
N2_bec-1 RNAi	Average	93,123	93,355	85,231	62,787	27,872	
	SEM	1,995	1,663	1,626	2,905	1,633	
	t-test vs N2			**	***		
		0,321350	0,245887	0,008487	0,000250	0,054125	
*atx-3(gk193)_*control RNAi	Average	90,626	91,068	95,272	67,730	0,313	
	SEM	1,939	2,313	1,529	3,587	0,292	
	t-test vs N2				**	***	
		0,079299	0,097113	0,317073	0,007093	0,0000001	
atx-3(gk193)_bec-1 RNAi	Average	97,462	93,442	79,658	25,505		
	SEM	4,427	7,088	3,700	8,093		
	t-test vs N2			**	***		
		0,808920	0,748880	0,008920	0,000010		
	t-test vs *atx-3*(*gk193*) control RNAi			**	***		
		0,206973	0,770172	0,002632	0,000453		

Average - Average survival for the designated time point (%)
SEM - Standard error of the mean
n = 8
* $p \leq 0.05$, ** $p \leq 0.01$, *** $p \leq 0.001$

Supporting Information Table S5: Statistical analysis for Figure S1A

		Day					
		1	5	8	11	14	16
N2_control RNAi	Average	90,760	84,174	79,087	80,942	49,752	22,269
	SEM	2,435	2,378	2,666	1,679	4,385	3,408
N2_unc-51 RNAi	Average	89,572	89,674	76,986	69,094	27,950	31,357
	SEM	1,333	2,740	1,675	2,817	3,812	1,635
	t-test vs N2				**	**	
		0,694815	0,178116	0,552923	0,004491	0,003464	0,097156
*atx-3(gk193)_* control RNAi	Average	94,346	82,238	76,928	77,378	34,343	41,383
	SEM	1,836	2,320	2,292	2,311	3,054	3,219
	t-test vs N2					*	**
		0,289923	0,594324	0,588235	0,262677	0,017336	0,001897
atx-3(gk193)_ unc-51-1 RNAi	Average	96,388	85,143	87,013	59,414	9,301	17,308
	SEM	1,140	2,223	3,965	1,926	1,203	2,991
	t-test vs N2				***	***	
		0,070460	0,784680	0,164410	0,000002	0,000001	0,323392
	t-test vs *atx-3*(*gk193*) control RNAi			*	***	***	***
		0,400661	0,211555	0,022775	0,000030	0,000001	0,000093

Average - Average survival for the designated time point (%)
SEM - Standard error of the mean
n = 8
* $p \leq 0.05$, ** $p \leq 0.01$, *** $p \leq 0.001$

Supporting Information Table S6: List of primers used for cloning the indicated constructs.

Final construct	Primer ID	Sequence
pEGFP-ATXN3 F1	F1 s	5’- AAT TAC TCG AGC TCA AAT GGA GTCC-3’,
	F1 as	5’-AAT AGG ATC CTC AAC CCC AAA CTT TCAA GG-3’
pEGFP-ATXN3 F2	F2 s	5’-AAT ACT CGA GCA TTA GAA CTA ATC-3’
	F2 as	5’-AAT AGG ATC CTC ACC TAA TCA TCT GCAG-3’
pEGFP-ATXN3 F3	F3 s	5’-AAT TAC TCG AGC AGT CCA ACA GAT GCAT-3’
	F3 as	5’-AAT AGG ATC CTC AGG CTT CTC GTC TCTT-3’
pEGFP-ATXN3 F4	F4 s	5’-AAT ACT CGA GCA TAC TTT GAA AAA CAG-3’
	F4 as	5’-CGG GGA TCC TTA TTT TTT TCC TTC TGT TTTC-3’
pEGFP-ATXN3 N-t. LIR1*	N.-t LIR1* s	5’-ATG GAT GAC AGT GGT GCT TTC TCT GCT CAG GTT ATA AGC AAT-3’
	N.-t LIR1* as	5’-ATT GCT TAT AAC CTG AGC AGA GAA AGC ACC ACT GTC ATC CAT-3’
pEGFP-ATXN3 LIR1*	LIR1* NEB s	5’-TAC AAG TCC GGA CTC AGA TCT CGA GCT CAA ATG GAG TCC ATC TTC-3’
	LIR1* NEB as	5’-ACT GTT GAA CAG GAT TAG TTC TAA ACC CC-3’
pEGFP-ATXN3 LIR2*	LIR2* s	5’-AAA TTA GGA AAA CAG GCG TTT AAC GCG AAT TCT CTC TTG ACG-3’
	LIR2* as	5’-CGT CAA GAG AGA ATT CGC GTT AAA CGC CTG TTT TCC TAA TTT-3’
pGEX-6P-1-ATXN3 (WT or LIRs)	GST-ATXN3 NEB s	5’-TCT GTT CCA GGG GCC CCT GGG ATC CAT GGA GTC CAT CTT CCA C-3’
	GST-ATXN3 NEB as	5’-GTC AGT CAC GAT GCG GCG CTC GAG TTA TTT TTT TCC TTC TGT TTTC-3’
GABARAP-GFP	GABARAP cds s	5’-CGG GAT CCG CCA GAC CGT AGA CACT-3’
	GABARAP cds as	5’-ACG CGT CGA CTC ATG AAG TTC GTG TAC-3’

Supporting Information Experimental Procedures

***Caenorhabditis elegans* maintenance and transgenic lines**

The following strains were used in this study: Bristol (N2) strain as wild-type (WT) strain, *cdc-48.1(tm544)II*, *cdc-48.1(tm544)II; atx-3(gk193)V, atx-3(gk193)V*, *atx-3(tm1689)V*, *atx-3(gk193)V; hhIs42[unc-119(+); atx-3::atx-3::GFP]*, *atx-3(gk193)V; hhIs43[atx-3::atx-3C20S::GFP; unc-119(+)]*, *rmIs133[unc- 54p::Q40::YFP]*, *atx-3(gk193)V; rmIs133[unc-54::Q40YFP]*. Some of the *C. elegans* strains were obtained from the Caenorhabditis Genetics Center (CGC). Mutant strains were outcrossed at least six times against the wild-type strain to provide isogenic conditions. Nematodes were grown at 20°C on nematode growth medium (NGM) agar plates seeded with *Escherichia coli* (*E. coli)* OP50 as food source (Brenner, 1974).

RNA Interference in worms

RNAi in *C. elegans* was performed using the RNAi feeding method (Timmons & Fire, 1998). In L1 survival assays, the maternal worm population was placed on HT115 *E. coli* bacteria expressing *bec-1*- or *unc-51*-specific double-stranded RNA (dsRNA) at L4 stage to obtain partial reduction of *bec-1* and *unc-51* expression in the progeny. After 24 hours of incubation worms were bleached with alkaline bleach solution, eggs were hatched at 20ºC overnight and L1 synchronized larvae starved as described for the starvation assay. As a control, bacteria transformed with the empty pPD129.36 vector (Fire Lab Vector Kit) were used as food source.

SDS-soluble aggregates

Worms expressing the aggregation-prone Q40::YFP protein were grown at 20ºC on NGM plates seeded with *E. coli* (OP50) until they reached adulthood (72h after L1 stage). Animals were washed off the plates with M9 buffer, settled on ice and washed in PBS containing protease inhibitor (Roche). Worms were lysed on ice using a Precellys homogenizer (2 x 20s 5,500 rpm) and sonicated (4 x 10s on ice; 60% power; Sonopuls UW 2200, Bandelin). Lysates were centrifuged at 4ºC for 15min at 15500 x g. Supernatant was kept as soluble (S) fraction. The pellet was washed once in PBS-lysis buffer and dissolved in SDS-lysis buffer (187.5 mM TrisHCl, 6% SDS, 30% sucrose) by sonication (4 x 10s (60%)). Samples were boiled in the presence of 50 mM DTT, followed by centrifugation at RT for 30 to 60min at 14,000 rpm. Supernatant was used as SDS-soluble (pellet, P) fraction.

Imaging of worms

Worms were anesthetized with 0.1 mM levamisole in M9 buffer, mounted on 3% agarose pads, and maintained at 20°C. Fluorescence mages were taken with an Axio Imager Z1 microscope equipped with ApoTome, EC-plan-Neofluar 10x/0.30 and EC-plan-Neofluar 40x/0.75 Air objectives, and Axiocam 503 mono camera (Carl Zeiss). Images were processed with Zen 2012 SP1 software (Carl Zeiss).

C. elegans immunoblotting and antibodies

Synchronized L1 larvae were grown on NGM-agar plates seeded with *E. coli* (OP50) until young adult (60h after L1 stage) day 1 adult stage (72h after L1 stage). Alternatively, chloroquine treated worms have been grown on NGM-agar plates seeded with *E. coli* (OP50) until L4 stage (48h after L1 stage), when they were moved on chloroquine-containing (concentration indicated in figure) NGM plates seeded with OP50 with for 24h prior to sample collection. Worm lysates used for SDS-PAGE were prepared by washing worms from NGM-agar plates followed by multiple washing step with M9 buffer, until bacteria were removed. Worm samples were taken up in SDS sample buffer followed by heating to 95°C for 5min and subsequently subjected to sonication (2x 30s, on ice; 60% power; Sonopuls UW 2200, Bandelin). Lysates were cleared by centrifugation at 8000 × g for 5min, and the supernatant was run on a 4-12% electrophoresis polyacrylamide gel containing sodium dodecyl sulfate (NuPAGE Novex 4-12% Bis-Tris Protein Gel, Invitrogen) and transferred to a nitrocellulose membrane (Bio-Rad Laboratories). Western blotting was performed using total worm lysates and antibodies against GFP (Mouse BD Living Colors A.v. Monoclonal Antibody (JL-8), Clontech) (1:5000 dilution), tubulin (Mouse Monoclonal anti-α-Tubulin, Sigma) (1:5000 dilution). For the detection of *C. elegans* ATX-3 protein, His-tagged purified ATX-3 was used for immunization of rabbits and anti-serum was affinity purified using GST-tagged recombinant ATX-3 protein (BioGenes) (1:20000 dilution) (Ackermann et al., 2016). Proteins were detected by ECL kit (GE Healthcare) using Peroxidase-conjugated AffiniPure Goat Anti-Mouse IgG+IgM (Jackson Immuno Research) secondary antibody (1:5000 dilution) or by Odyssey® CLx Infrared Imaging System (LI-COR) using Li-Cor donkey anti-mouse or anti-rabbit IRDye 800CW/680 antibodies (1:10000 dilution). Immunoblots shown in the figures are representative of at least 2 independent experiments, unless otherwise indicated.

Cell culture

HeLa, U2OS, HOS green fluorescent protein (GFP)-LC3 (Eng, Panas, Karlsson Hedestam, & McInerney, 2010), U2OS monomeric red fluorescent protein (mRFP)-GFP-LC3, mouse

embryonic fibroblasts (MEFs) ATXN3 WT/WT and KO/KO (Schmitt et al., 2007; Weishaupl et al., 2019) and HeLa tet-on ^{10xHis}ATXN3^{HA} (Pfeiffer et al., 2017) cells were cultured in DMEM+GlutaMAX (Life Technologies) supplemented with 10% Fetal Bovine Serum (FBS) or 10% FBS Performance Plus (Life Technologies) in a humidified chamber at 37˚C and 5% CO_2. Expression of ^{10xHis}ATXN3^{HA} was induced by addition of 1 µg/mL doxycycline for 24h. To induce autophagy, cells were starved in Earle's Balanced Salt Solution (EBSS; Life Technologies) for 2 or 4h. Autophagy was blocked using of 100 nM Wortmannin (Sigma), 10 mM 3-methyladenine (3-MA; Sigma) or 100 nM BafilomycinA1 (Vivabioscience). Plasmid DNA and siRNA were transfected using Lipofectamine 2000 or Lipofectamine3000 (LifeTechnologies) according to the manufacturer's instructions.

Plasmids

The pEGFP-ATXN3 wild-type, pEGFP-ATXN3 C14A, pEGFP-ATXN3 N-terminus (N-t.), pEGFP-ATXN3 C-terminus (C-t.), mCherry-ATXN3 wild-type and the pGEX-ATXN3 constructs have been described previously (Pfeiffer et al., 2017). mCherry-ATXN3 C14A was generated by replacing wild-type ATXN in the mCherry-C1 vector with ATXN3 C14A obtained from the pEGFP-ATXN3 C14A vector by *BamH*I/*Xho*I restriction digest and ligation. pEGFP-ATXN3 truncations F1 (1-88), F2 (89-182), F3 (183-287) and F4 (288-361) were generated by PCR amplification of the respective fragments using the primers listed in Supplementary Table 1 and cloned into the pEGFP-C1 backbone using the *Xho*I/*Bam*HI restriction sites. The pEGFP-ATXN3 N-t. LIR1* (^{72}SGFFSI to ^{72}SGAFSA) mutant was generated by site-directed mutagenesis (Quick-Change II, Site-directed Mutagenesis Kit, Agilent; **Supporting Information Table S6**) of pEGFP-ATXN3 N-t. Based on this mutant, pEGFP-ATXN3 (FL) LIR1* was generated by Gibson assembly using the NEBuilder HiFi DNA Assembly Cloning Kit (New England BioLabs) according to the manufacturer's instructions. For this, the pEGFP-ATXN3 was used as backbone and linearized by restriction digest with *Xho*I/*Pst*I. LIR1* mutant insert was PCR amplified using the primers listed in Supplementary Table S1. pEGFP-ATXN3 LIR2* (^{128}KQWFNL to ^{128}KQAFNA) and the pEGFP-ATXN3 LIR1*/2* double mutant were generated by site directed mutagenesis (Quick-Change II, Site-directed Mutagenesis Kit, Agilent) of pEGFP-ATXN3 and pEGFP-ATXN3 LIR1*, respectively. The pGEX-ATXN3 LIR mutants were created by Gibson assembly using the NEBuilder HiFi DNA Assembly Cloning Kit (New England BioLabs) according to the manufacturer's instructions. The pGEX-6P-1 was linearized by restriction digest using *Bam*HI/*Xho*I and ATXN3 WT or LIR mutants PCR amplified from the respective pEGFP-

ATXN3 vector (**Supporting Information Table S1**). pGEX-GABARAP-GFP was generated by PCR amplification (**Supporting Information Table S1**) of GABARAP cDNA and ligated into the pEGFP-N1 vector using *Sal*I/*Bam*HI restriction sites. GABARAP-GFP was subcloned into the pGEX-6P-1 vector using *Sal*I/*Not*I restriction sites. Bacterial expression vectors pGEX-5X-3 (GST control), pDEST15-LC3A, pDEST15-LC3B, pDEST15-LC3C, pDEST15-GABARAP, pDEST15-GABARAPL1, pDEST15-GABARAPL2 were previously described (Lystad et al., 2014).

RNA interference

The following siRNA oligonucleotides were used at a final concentration of 30 nM in this study: AllStars negative control siRNA (Qiagen); ATXN3 #1, 5'-GCA CUA AGU CGC CAA GAA A-3'; ATXN3 #2, 5'-ACG AAG AUG AGG AGG AUU U-3' (custom siRNA oligonucleotides, Dharmacon); p97, 5'-AAC AGC CAU UCU CAA ACA GAA-3' (custom siRNA oligonucleotides, Ambion).

GFP-LC3 puncta assay

For analysis of living cells, HOS-GFP LC3 cells were plated, transfected and treated in a 96-well imaging plate (BD Falcon™). Hoechst (2 µg/mL in 1 x PBS) was added to the cells and incubated for 30min. Prior to imaging, medium was replaced by Leibovitz's L-15 medium (Life Technologies) and four to six sites per well imaged without delay using an ImageXpress automated widefield microscope (Molecular Devices) equipped with a 20 x objective. Analysis of immunolabeled cells was done similarly. The number of cytoplasmic GFP-LC3 puncta per cell was quantified using CellProfiler Software 2.1.1. (Broad Institute). For rescue experiments, cytoplasmic GFP-LC3 puncta were quantified in mCherry-positive cells. The threshold for classification as mCherry-positive was set as higher than the mean intensity plus two times the standard deviation ($> mean + 2 * SD$) of mock-transfected HOS GFP-LC3 cells.

Flow cytometry analysis of autophagic flux

For analysis of autophagic flux by flow cytometry, U2OS mRFP-GFP-LC3 cells were cultured, transfected and treated in 6-well plates. Cells were harvested with trypsin and washed with PBS. Extraction of non-autophagosome associated mRFP-GFP-LC3 was done by briefly washing with 0.05% saponin in PBS (Eng et al., 2010). Using the BD FACSAria III, 30000 events were captured per experiment and cellular mRFP- and GFP-intensities measured using the 488nm and 561nm laser. Data were analyzed using FlowJo software (Treestar Inc.).

Autophagic flux was determined as the ratio between mean intensities of mRFP and GFP and normalized to Bafilomycin A1-treated, starved controls in four independent experiments.

Immunofluorescent labelling

Cells were cultured on 18 mm coverslips or in 96-well imaging plates and transfected as described above. Cells were fixed in 4% paraformaldehyde in 1 x phosphate-buffered saline (PBS) for 15 min at room temperature or 30 min on ice. Cells were permeabilized using 0.2 % Triton X-100 or 0.2 % saponin in 1 x PBS for 15 min and incubated with 100 mM glycine in 1 x PBS for 10 min at room temperature. Unspecific binding was blocked using 3% BSA in 1 x PBS for 30 min at RT. Primary antibodies were diluted in 1 x PBS containing 0.1% Tween-20 (PBS-T): mouse anti-c-Myc 1:100 (Santa Cruz, 9E10, sc-40), mouse anti-HA 1:100 (Covance, 16B12, MMS-101R), rabbit anti-HA 1:100 (Abcam, ab9110), mouse anti-p97/VCP 1:1000 (Abcam, ab11433), mouse anti-WIPI2 1:200 (Abcam, ab105459), rabbit-anti LC3B (MBL Life Science, PM036) . Goat anti-mouse or goat anti-rabbit IgG coupled to Alexa 488, 546 or 647 were diluted 1:500 in PBS-T. Nuclear staining was performed using Hoechst (2 µg/mL in 1 x PBS) and samples mounted using Mowiol. Confocal images were acquired using the Zeiss LSM510 META or LSM710. Images were analysed using LSM Image Browser. Line scans were performed using ImageJ. The percentage of ^{3xMyc}ATG16L1 and GFP-LC3 puncta that were associated with or colocalizing with^{10xHis}ATXN3^{HA} were quantified manually.

Western blotting

Cells were harvested in 2 x LDS sample buffer containing 2 x NuPAGE reducing agent (Life Technologies), extracts boiled at 95˚C for 5min and separated by SDS-PAGE (NuPAGE 4-12% Bis-Tris protein gels, NuPAGE 12% Bis-Tris protein gels), followed by transfer onto PVDF or Nitrocellulose membranes. Membranes were blocked in Tris-buffered saline (TBS) containing 5 % skimmed milk (TBS-Milk). Immunoblotting was performed using the following antibodies diluted in TBS-Milk: mouse anti-ataxin-3 1:1000 (Millipore, 1H9, MAB530), rabbit anti-ataxin-3 1:1000 (Novus Biological, NBP32083), rabbit anti-ATG16L1 1:1000 (MBL Life Science, PM040), anti-β-actin 1:5000 (Sigma, AC-74, A2228), rabbit anti-beclin-1 1:1000 (Cell Signaling Technology, CS3738), rabbit anti-GABARAP 1:1000 (MBL Life Science, PM037), rabbit anti-GAPDH 1:2500 (Abcam, ab9485), rabbit anti-GFP 1:5000 (Abcam, ab290), mouse anti-His-tag 1:2000 (MBL Life Science, JM-3646-100), rabbit anti-LC3B 1:2000 (Sigma, L7543), mouse anti-p62 1:500 (BD Bioscience, BD610832), mouse anti-p97/VCP 1:5000 (Abcam, ab11433), mouse anti-RFP 1:2500 (used to detect mCherry; Chromotek, 6G6). HRP-

conjugated sheep anti-mouse IgG (GE Healthcare, NXA931V) and sheep anti-rabbit IgG (GE Healthcare, NXA934V), 1:5000 were used, followed by detection using Amersham ECL Reagent (Ge Healthcare) on Medical XRay films (Fuji).

Protein purification

Purification of recombinant GST-tagged or untagged proteins was done as previously described (Pfeiffer et al., 2017). Briefly, GST-fusion proteins were expressed in BL21 *E. coli* and purified using Glutathione sepharose beads in GST-lysis buffer (50 mM HEPES pH 7.5, 500 mM NaCl, 10% glycerol, 1 mM MgCl2, 10 mM DTT). To obtain untagged proteins, protein was eluted using PreScission protease (120 U/mL; GE Healthcare).

Atg8 interaction

LC3A- or GABARAP-agarose beads or unconjugated agarose beads (negative control) were equilibrated in Buffer A (150 mM NaCl, 50 mM Tris pH 7.5, 1 mM DTT, 0.5 mM EDTA, 0.5% NP-40). 1 µg purified, recombinant ataxin-3 was added to the beads and incubated for 1.5 h at 4˚C. Beads were washed five times and eluted in 2 x LDS sample buffer containing 2 x NuPAGE reducing agent. For Atg8 interaction in cell lysates, cells were lysed on ice for 30min in 1 x NP-40 lysis buffer (150 mM NaCl, 10 mM Tris pH 7.5. 0.5 mM EDTA, 0.5 % NP-40, 1 x complete EDTA-free protease inhibitor cocktail (Roche Applied Science), 20 mM NEM (Sigma-Aldrich), 10 µM MG132 (Enzo)). Lysates were cleared at 16,000 x g for 10min at 4˚C and incubated with 20 µL empty agarose beads (negative control) or 20 µL recombinant LC3A- or GABARAP-agarose (BostonBiochem, UL-435, UL-415) for 2h at 4 ˚C. Beads were washed twice in 1 x NP-40 lysis buffer, followed by three times in wash buffer (150 mM NaCl, 10 mM Tris pH 7.5. 0.5 mM EDTA, 1 x protease inhibitor cocktail, 20 mM NEM, 10 µM MG132) and eluted in 2 x LDS sample buffer containing reducing agent and boiled at 95˚C for 5min.

GST-pulldown assay

GST-tagged fusion proteins were expressed in *E. coli* BL21 (DE3) Star and purified with Glutathione Sepharose (GE Healthcare Bio-Sciences AB). Cell extracts from tetracycline induced HeLa tet-on ^{10xHis}ATXN3^{HA} cells were mixed with GST-tagged proteins, bound to Glutathione Sepharose, and incubated in lysis buffer (1% Triton X-100, 50mM Tris pH 7.5, 50mM NaCl, 5mM EDTA) supplemented with complete EDTA-free protease inhibitor cocktail (Roche Applied Science)) for 1 h at 4°C, washed five times with lysis buffer, boiled with 2×

LDS sample buffer containing 2 x NuPAGE reducing agent, and analysed by SDS-PAGE and immunoblotting with GST and HIS antibody using a Licor Odyssey imager.

Cleavage of preGABARAP-GFP

Recombinant preGABARAP-GFP (250 ng) was incubated with 0.1 µM or 1 µM recombinant ataxin-3 or 0.1 µM recombinant Atg4B or in the absence of protease in DUB buffer (50 mM HEPES pH 8, 0.5 mM EDTA, 1 mM DTT, 0.1 µg/ml BSA) for 16h. The reaction was stopped by addition of 2 x LDS sample buffer containing 2 x NuPAGE reducing agent, samples were boiled at 95˚C and analysed by SDS-PAGE and immunoblotting.

DUB assay

Ataxin-3 DUB-activity was assessed as previously described (Pfeiffer et al., 2017). Briefly, GST-ataxin-3 was incubated with 0.25 µg hexa-K63-ubiquitin (BostonBiochem, UL-317, in 50 mM Tris pH 7.5) in DUB buffer (50 mM HEPES pH 8, 0.5 mM EDTA, 1 mM DTT, 0.1 µg/ml BSA) for 16h at 37°C. The reaction was stopped by addition of 2 x LDS sample buffer containing 2 x NuPAGE reducing agent and samples analysed by SDS-PAGE and immunoblotting.

***In silico* analysis of putative LIR motifs**

In silico analysis of putative LIR motifs in the sequence of ataxin-3 was done using the iLIR prediction tool (http://repeat.biol.ucy.ac.cy/iLIR/) (Kalvari et al., 2014).

Statistical analysis

Statistical analysis was performed using GraphPad Prism 6. Gaussian distribution of data was tested using the Shapiro-Wilk normality test. Data with normal distribution are presented as mean ± standard deviation (SD), unless stated differently, and were analysed using unpaired Student's t-test (to compare two groups) or one-way ANOVA (to compare multiple groups) followed by Bonferroni to either compare all pairs or selected pairs. Non-normally distributed data are shown as box plots with median and 5-95 percentile and were analysed using the non-parametric Kruskal-Wallis test followed by Dunn's to compare multiple groups or selected pairs. Data shown are generated from at least three independent experiments (if not stated differently in figure legends). For data obtained with worms, mean values ± SEM are shown, unless otherwise indicated. Statistical comparisons between groups were done using two-tailed

Student's t test. Quantification of immunoblots was done with ImageJ 1.48v or Image Studio 4.0 software. Data are presented as fold change compared to the control. The following p-values were considered significant: * $p \leq 0.05$, ** $p \leq 0.01$, *** $p \leq 0.001$. Statistical significance was assessed by two-tailed paired student's t-test using Excel software (Microsoft) or GraphPad Prism 6.

References

Ackermann, L., Schell, M., Pokrzywa, W., Kevei, E., Gartner, A., Schumacher, B., & Hoppe, T. (2016). E4 ligase-specific ubiquitination hubs coordinate DNA double-strand-break repair and apoptosis. *Nat Struct Mol Biol, 23*(11), 995-1002. doi:10.1038/nsmb.3296

Brenner, S. (1974). The genetics of Caenorhabditis elegans. *Genetics, 77*(1), 71-94.

Eng, K. E., Panas, M. D., Karlsson Hedestam, G. B., & McInerney, G. M. (2010). A novel quantitative flow cytometry-based assay for autophagy. *Autophagy, 6*(5), 634-641.

Kalvari, I., Tsompanis, S., Mulakkal, N. C., Osgood, R., Johansen, T., Nezis, I. P., & Promponas, V. J. (2014). iLIR. *Autophagy, 10*(5), 913-925. doi:10.4161/auto.28260

Lystad, A. H., Ichimura, Y., Takagi, K., Yang, Y., Pankiv, S., Kanegae, Y., . . . Simonsen, A. (2014). Structural determinants in GABARAP required for the selective binding and recruitment of ALFY to LC3B-positive structures. *Embo Reports, 15*(5), 557-565. doi:10.1002/embr.201338003

Pfeiffer, A., Luijsterburg, M. S., Acs, K., Wiegant, W. W., Helfricht, A., Herzog, L. K., . . . Dantuma, N. P. (2017). Ataxin-3 consolidates the MDC1-dependent DNA double-strand break response by counteracting the SUMO-targeted ubiquitin ligase RNF4. *Embo J, 36*(8), 1066-1083. doi:10.15252/embj.201695151

Schmitt, I., Linden, M., Khazneh, H., Evert, B. O., Breuer, P., Klockgether, T., & Wuellner, U. (2007). Inactivation of the mouse Atxn3 (ataxin-3) gene increases protein ubiquitination. *Biochem Biophys Res Commun, 362*(3), 734-739. doi:10.1016/j.bbrc.2007.08.062

Timmons, L., & Fire, A. (1998). *Specific interference by ingested dsRNA*: Nature. 1998 Oct 29;395(6705):854. doi: 10.1038/27579.

Weishaupl, D., Schneider, J., Peixoto Pinheiro, B., Ruess, C., Dold, S. M., von Zweydorf, F., . . . Schmidt, T. (2019). Physiological and pathophysiological characteristics of ataxin-3 isoforms. *J Biol Chem, 294*(2), 644-661. doi:10.1074/jbc.RA118.005801
